# Genomic Prediction of Adaptation in Common Bean (*Phaseolus vulgaris* L.) × Tepary Bean (*P. acutifolius* A. Gray) Hybrids

**DOI:** 10.3390/ijms26157370

**Published:** 2025-07-30

**Authors:** Felipe López-Hernández, Diego F. Villanueva-Mejía, Adriana Patricia Tofiño-Rivera, Andrés J. Cortés

**Affiliations:** 1 Corporación Colombiana de Investigación Agropecuaria (AGROSAVIA)—CI La Selva, Km 7 vía Rionegro—Las Palmas, Rionegro 054048, Colombia; llopez@agrosavia.co; 2 Applied Sciences and Engineering School, EAFIT University, Medellín 050022, Colombia; dvillanu@eafit.edu.co; 3 Corporación Colombiana de Investigación Agropecuaria (AGROSAVIA)—CI Motilonia, Codazzi 478020, Colombia; atofino@agrosavia.co; 4 Facultad de Ciencias Agrarias-Departamento de Ciencias Forestales, Universidad Nacional de Colombia-Sede Medellín, Medellín 050034, Colombia; 5 Department of Plant Breeding, Swedish University of Agricultural Sciences, 23436 Lomma, Sweden

**Keywords:** heat tolerance, introgression breeding, Bayesian modeling, northwest South America, humid and dry coastal Colombia, genotyping by sequencing (GBS)

## Abstract

Climate change is jeopardizing global food security, with at least 713 million people facing hunger. To face this challenge, legumes as common beans could offer a nature-based solution, sourcing nutrients and dietary fiber, especially for rural communities in Latin America and Africa. However, since common beans are generally heat and drought susceptible, it is imperative to speed up their molecular introgressive adaptive breeding so that they can be cultivated in regions affected by extreme weather. Therefore, this study aimed to couple an advanced panel of common bean (*Phaseolus vulgaris* L.) × tolerant Tepary bean (*P. acutifolius* A. Gray) interspecific lines with Bayesian regression algorithms to forecast adaptation to the humid and dry sub-regions at the Caribbean coast of Colombia, where the common bean typically exhibits maladaptation to extreme heat waves. A total of 87 advanced lines with hybrid ancestries were successfully bred, surpassing the interspecific incompatibilities. This hybrid panel was genotyped by sequencing (GBS), leading to the discovery of 15,645 single-nucleotide polymorphism (SNP) markers. Three yield components (yield per plant, and number of seeds and pods) and two biomass variables (vegetative and seed biomass) were recorded for each genotype and inputted in several Bayesian regression models to identify the top genotypes with the best genetic breeding values across three localities on the Colombian coast. We comparatively analyzed several regression approaches, and the model with the best performance for all traits and localities was BayesC. Also, we compared the utilization of all markers and only those determined as associated by a priori genome-wide association studies (GWAS) models. Better prediction ability with the complete SNP set was indicative of missing heritability as part of GWAS reconstructions. Furthermore, optimal SNP sets per trait and locality were determined as per the top 500 most explicative markers according to their *β* regression effects. These 500 SNPs, on average, overlapped in 5.24% across localities, which reinforced the locality-dependent nature of polygenic adaptation. Finally, we retrieved the genomic estimated breeding values (GEBVs) and selected the top 10 genotypes for each trait and locality as part of a recommendation scheme targeting narrow adaption in the Caribbean. After validation in field conditions and for screening stability, candidate genotypes and SNPs may be used in further introgressive breeding cycles for adaptation.

## 1. Introduction

Food insecurity is still a major issue in modern societies [1], with at least 713 million people facing hunger [2]. This scenario is aggravated in vulnerable localities of the world, such as Latin America and the Caribbean, where in 2020 47.7 million people lived with hunger [3], a figure that in 2022 increased to 56.5 million people [2]; additionally, there were 93.5 million people living with food insecurity [2]. Fortunately, legumes offer a nature-based solution to tackle food insecurity, as they are a source of nutrients for rural communities in Latin America and Africa thanks to their high content of nutrients, proteins, and dietary fiber [4,5]. Among legume species, the common bean (*Phaseolus vulgaris* L.) is one of the most planted with ~27 million tons worldwide, China and America being the leading producers [6].

Climate change is further aggravating the food security challenge for humanity because producing enough food for the growing world population is being limited by extreme heat and drought events [7]. The Caribbean region in particular appears negatively impacted by climate change, with projections of a 3.75% decrease in average precipitation and a 0.76 °C increase in temperature by 2050 [8,9]. Despite the common bean being a crucial food security component and part of the cultural heritage of communities on the Caribbean coast of northwest South America, it remains heat and drought susceptible [10]. Therefore, the current climate change scenario is limiting bean productivity in the Caribbean and overall food security in the region, making it imperative to speed up the molecular breeding of common beans.

Breeding crops for climate change adaptation requires targeting reservoirs of abiotic stress tolerance [11,12], involving pre-breeding phases capable of mobilizing the adaptative potential to crop lines in extreme environments. Modern genomic resources, such as genotyping by sequencing (GBS), and analytical approaches, like GIS-based modeling and machine learning (ML) algorithms, have also assisted in this endeavor [11]. Unfortunately, introgressive breeding of adaptative variation empowered by molecular markers has limitedly been deployed for common beans in climate-vulnerable developing regions, such as the Colombian Caribbean [13]. The above despite genetic resources from closely related *Phaseolus* species may leverage natural variation for adaptation to abiotic stresses, like heat and drought [14]. Specifically, the Tepary bean (*P. acutifolius* A. Gray) is a bean native to northwest Mexico that was domesticated near the arid border with the USA [15,16]. The adaptation of the Tepary bean to hot [17] and dry environments [18,19] makes it the most heat-tolerant species of the *Phaseolus* genus. Yet, the Tepary bean is limited as a modern crop compared to the more susceptible but commercially accepted common bean. Consequently, a more feasible alternative would be to use the Tepary bean as an exotic donor of adapted alleles [20] to improve drought and heat tolerance in the common bean [21]. Despite interspecific incompatibilities, common beans have already been backcrossed with Tepary donors with a relatively good viability rate using bridge genotypes [22,23]. We have also recently explored the complex polygenetic architecture of yield components and biomass variables of common bean × Tepary bean interspecific advanced lines, suggesting that the genetic basis of adaptation is polygenic and differs across the dry and humid Caribbean sub-regions [24]. Nonetheless, since polygenic adaptation is environmentally dependent in hybrid beans [24], conventional phenotypic-based backcrossing would lack sufficient power for allelic augmentation.

An alternative is offered by multi-locality genomic prediction (GP), an analytical innovation that merges quantitative genetics theory with genomics [2,25,26]. GP calibrates marker-based infinitesimal additive predictive models [27,28,29] using robust phenotypic data [30], usually for quantitative polygenic traits. For the calibration of GP models, a wide range of statistical algorithms have been proposed [31]. The parametric approach most widely implemented in GP is regularized regression, which uses partial or whole-genome regression to tackle the high-dimensionality and multicollinearity with optimization in the parameter estimation by restricted maximum likelihood (REML) or Bayesian approaches [3,32]. Nonparametric approaches are also utilized, such as the Reproducing Kernel Hilbert Spaces (RKHS) regression [33]. To further optimize the number of molecular markers used in GP and escalate their implementation to more ambitious panels of genotypes, authors have explored the use of reduced subsets of single nucleotide polymorphism (SNPs) markers capable of maintaining prediction abilities [29,34,35]. Associated SNPs from a priori genome-wide association studies (GWAS) may offer such an opportunity to pre-select or preferentially weight more explicative markers within the inputted genomic datasets [36,37].

Since we previously found that the polygenic adaptation in terms of yield and biomass variables in hybrid genotypes of beans is environmentally dependent across localities [24], now we wonder how the genomic prediction ability and heritability scores would vary in the Caribbean region using the advanced panel of common bean (*P. vulgaris* L.) × Tepary bean (*P. acutifolius* A. Gray) interspecific lines. With this question in mind, the objectives of this study were to (1) estimate the genomic heritability and prediction ability for yield and biomass traits across three localities in the Colombian Caribbean and (2) evaluate via comparative analysis the accuracy of GP using a battery of modern algorithms and two genotyping setups: all markers from GBS and the associated markers from previous genome-wide association studies (GWAS) models [24,38]. Identifying the top genotypes using the genomic estimated breeding values (GEBVs) in a bean panel with interspecific Tepary ancestries would aid indirect selection and speed up the breeding of common bean varieties targeting extreme climatic conditions in terms of heat and drought, such as those found in coastal Colombia.

## 2. Results

The prediction ability of all models tended to be higher using all markers than using only the associated markers for all yield components and biomass variables across localities. Also, in all models, the mean square error was lower for the training sets than the testing sets, as expected. The model screening suggested that BayesC was the model with the best performance across all localities in all yield components and biomass variables using all markers. Despite the genomic heritability differing among localities, the predictive ability was generally constant using all markers. Most ad hoc predictive SNPs suggested specific SNP-chips for each locality and trait, while GEBV estimates enabled recommending superior interspecific genotypes for future breeding cycles.

### 2.1. Yield Components and Biomass Variables Were Correlated and Differed Across Localities

Almost all correlations among yield components and biomass variables were significantly positive (*p*-values_Bonferroni_ < 0.05, Figure S2). The significance was comparable among the parametric and nonparametric approaches (Figure S2A,B). Yet, the correlation scores differed among localities (Figure S2C,D). The correlations between the number of pods (NP) and yield (YLP) were moderate between Turipaná and Motilonia localities and the lowest in Carmen de Bolívar (Figure S2C,D). Also, the correlations among vegetative biomass (VB), seed biomass as per seed weight (SB), and the number of pods (NP) were higher in Motilonia than in Carmen de Bolívar (Figure S2C,D).

### 2.2. BayesC Was the Model with the Best Performance Across Traits and Localities

Predictive ability, heritability, and squared error scores are summarized in Table S2. Genomic prediction modeling with all markers showed that the approach with the highest predictive ability was the BayesC algorithm for all yield components, biomass variables, and localities (YLP in Figure S3, NS in Figure S4, NP in Figure S5, SB in Figure S6, and VB in Figure S7). However, the prediction ability across all models was reduced when relying only on the associated markers instead of the full marker set. There were no significant differences among the Bayesian alphabet models (BayesA, BayesB, and BayesC) as per Kruskal’s test of median differences; yet in some cases, the predictive ability differed between the Bayesian alphabet and the BL, BRR, and RKHS models. At the Motilonia locality, all GP modeling approaches had a more homogeneous performance without significant differences, except for the BayesC and BL algorithms (P_Bonferroni_ = 0.02).

In terms of predictive ability for YLP, GP modeling with BayesC using all markers had the highest performance, with a median prediction ability of 0.83 (±0.17) in Carmen de Bolivar, 0.81 (±0.10) in Motilonia, and 0.79 (±0.10) in Turipaná. In Carmen de Bolivar, the Kruskal test suggested significant differences between the model RKHS and the models BayesA (P_Bonferroni_ = 0.01), BayesB (P_Bonferroni_ = 0.02), and BayesC (P_Bonferroni_ = 3.03 × 10^−4^). In Turipaná, significant differences were observed between the model BayesC and the models BRR (P_Bonferroni_ = 0.04) and RKHS (P_Bonferroni_ = 4.53 × 10^−4^). For NS, GP modeling with BayesC using all markers had the highest performance, with a median prediction ability of 0.83 (±0.18) in Carmen de Bolivar, 0.81 (±0.09) in Motilonia, and 0.85 (±0.08) in Turipaná. In Carmen de Bolivar, the Kruskal test suggested significant differences between the model BayesC and the models BL (P_Bonferroni_ = 0.02), BRR (P_Bonferroni_ = 0.042), and RKHS (P_Bonferroni_ = 2.01 × 10^−4^). In addition, the model RKHS presented significant differences with the models BayesA (P_Bonferroni_ = 6.09 × 10^−3^) and BayesB (P_Bonferroni_ = 0.01). In Turipaná, significant differences were captured between the BayesC model with the models BL (P_Bonferroni_ = 0.03), BRR (P_Bonferroni_ = 6.95 × 10^−3^), and RKHS (P_Bonferroni_ = 7.80 × 10^−5^). Additionally, the Kruskal test suggested significant differences between RKHS and BayesA (P_Bonferroni_ = 0.03). For NP, GP modeling with BayesA using all markers had the highest performance, with a median prediction ability of 0.75 (±0.10) in Carmen de Bolivar. However, BayesC was the model with the highest performance in Motilonia and Turipaná, with a median prediction ability of 0.82 (±0.10) and 0.84 (±0.13), respectively. In Carmen de Bolivar, significant differences were observed between the model RKHS and BayesA (P_Bonferroni_ = 0.01) and BayesC (P_Bonferroni_ = 0.03). In Turipaná, the model BayesC presented significant differences with the models RKHS (P_Bonferroni_ = 7.54 × 10^−5^), BRR (P_Bonferroni_ = 2.62 × 10^−3^), and BL (P_Bonferroni_ = 0.02), in addition to significant differences between RKHS and the model BayesA (P_Bonferroni_ = 0.01). For SB, GP modeling with BayesC using all markers showed the highest performance, with a median prediction ability of 0.84 (±0.17) in Carmen de Bolivar and 0.83 (±0.10) in Motilonia. In Carmen de Bolivar, the Kruskal test suggested significant differences between the model BayesC and BL (P_Bonferroni_ = 0.01), BRR (P_Bonferroni_ = 0.02), and RKHS (P_Bonferroni_ = 1.49 × 10^−3^), in addition to significant differences between the model RKHS and BayesB (P_Bonferroni_ = 0.04) and BayesA (P_Bonferroni_ = 0.03). Finally, for the VB index, GP modeling with BayesA using all markers had the highest performance, with a median prediction ability of 0.75 (±0.10) in Carmen de Bolivar, but BayesC and BayesA were the models with the highest performance, with a median prediction ability of 0.83 (±0.08 and SD = 0.10, respectively) in Motilonia. In Carmen de Bolivar, the Kruskal test suggested significant differences between the model RKHS and the models BayesC (P_Bonferroni_ = 0.02) and BayesA (P_Bonferroni_ = 3.75 × 10^−3^).

On the other hand, the Bayes alphabet had the lowest mean squared error in the testing sets for each yield component and biomass variable. For YLP, BayesC had the lowest mean squared error (MSE_Carmen_ = 0.038, MSE_Motilonia_ = 0.010, MSE_Turipana_ = 0.093) in the testing dataset across all models and localities using all markers (Figure S8). For the NS index, BayesC had the lowest mean squared error (MSE_Carmen_ = 0.037, MSE_Motilonia_ = 0.010, MSE_Turipana_ = 0.097) in the testing dataset across all models and markers (Figure S9). Similarly, for the NP index using all markers, the models with the lowest mean squared error in the testing dataset were BayesA in Carmen de Bolivar (MSE_Carmen_ = 0.064) and BayesC in Motilonia and Turipaná (MSE_Motilonia_ = 0.015, MSE_Turipana_ = 0.033) (Figure S10). For the SB index, BayesC had the lowest mean squared error (MSE_Carmen_ = 0.030, MSE_Motilonia_ = 0.018) in the testing dataset across all models and markers (Figure S11). Additionally, for the VB index, BayesC had the lowest mean squared error (MSE_Carmen_ = 0.018, MSE_Motilonia_ = 0.018) in the testing dataset for all models and markers (Figure S12).

Meanwhile, the prediction ability across all models tended to exhibit reduced performance when relying only on the a priori GWAS-associated markers compared to all markers (Figures S3–S7). Also, the Kruskal test did not report any significant difference in terms of performance among models across localities for the yield components and biomass variables using only associated markers. In this sense, we selected the model BayesC for the following analyses from all the Bayes alphabet because this approach had the highest performance and the lowest mean squared error (Table 1).

### 2.3. All Markers Conveyed Greater Precision and Heritability than Only Associated Markers

**Figure 1:**
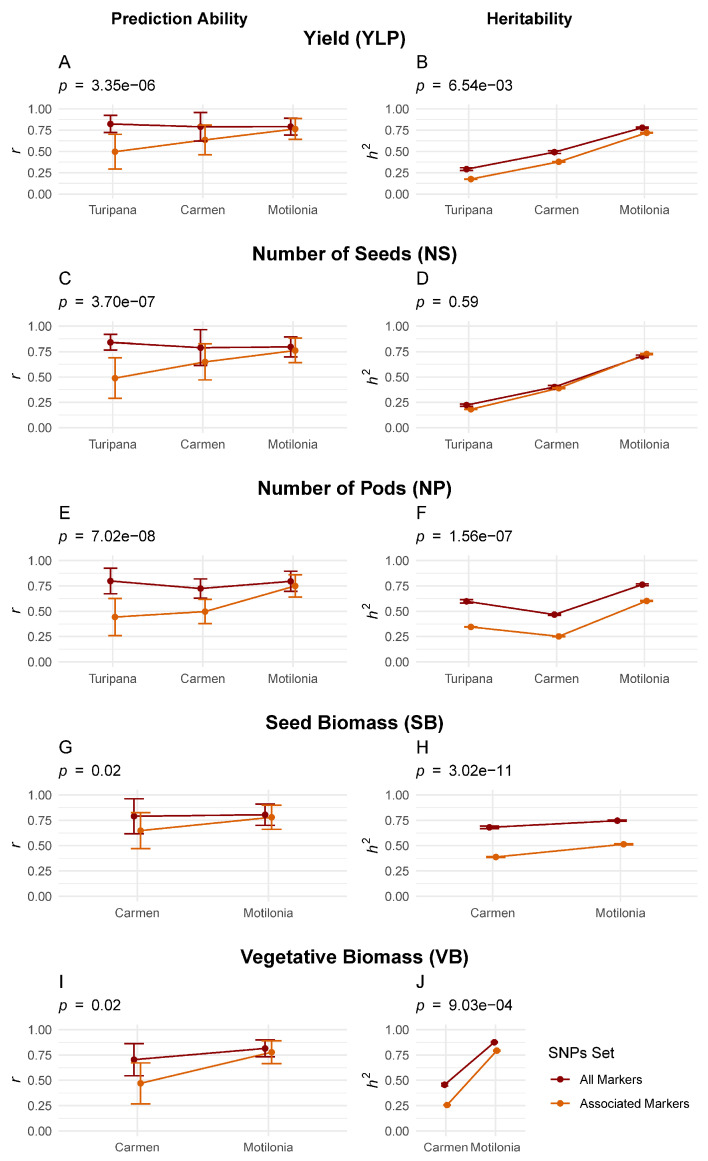
Prediction ability and heritability for five yield components and biomass variables across three localities as per BayesC. One-way ANOVA with the Mann–Whitney U tested differences between the two SNP sets (colored lines: all markers *vs.* GWAS-associated markers) using the R-Package *ggstatsplot* [39]. (**A**) Prediction ability and (**B**) heritability of yield per plant (YLP). (**C**) Prediction ability and (**D**) heritability of the number of seeds per pod (NS). (**E**) Prediction ability and (**F**) heritability of the number of pods (NP). (**G**) Prediction ability and (**H**) heritability of seed biomass (SB) measured as seed weight. (**I**) Prediction ability and (**J**) heritability of vegetative biomass (VB).

For all yield components and biomass variables, the heritability and prediction ability scores calculated by BayesC using all markers were significantly higher than those obtained only from GWAS-associated markers (Figure 1). For all yield components and biomass variables, the heritability scores in Motilonia (*h*^2^ > 0.75) were higher than the ones obtained in the other localities using all markers or only the associated markers, except for NS in Motilonia (Table 1, Figure 1D, *p*-value = 0.59). Yet, heritability estimates in Motilonia were equivalent when using all markers or only associated ones (Figure 1).

### 2.4. Genomic Heritability Differed Among Localities While Predictive Ability Was Consistent

Based on the Mann–Whitney U test, predictions were better in the dry localities than in the humid research station (Figure 1) when relying only on the associated markers for YLP (*p*-value = 3.96 × 10^−4^), NS (*p*-value = 3.11 × 10^−4^), NP (*p*-value = 1.34 × 10^−5^), SB (*p*-value = 0.02), and VB (*p*-value = 8.13 × 10^−5^). However, the predictions were generally constant across the localities when all markers were used for YLP (*P_value_* = 0.74), NS (*p*-value = 0.52), NP (*p*-value = 0.03), SB (*p*-value = 0.93), and VB (*p*-value = 0.04). Also, heritability scores were significantly higher across all localities when using all markers as compared to estimates that only relied on the associated markers for YLP (*p*-value =4.49 × 10^−3^), NS (*p*-value = 0.38), NP (*p*-value = 7.56 × 10^−46^), SB (*p*-value = 1.64 × 10^−69^), and VB (*p*-value = 2.17 × 10^−3^). On the other hand, the greatest missing heritability ( $h_{m\;}^{2}$ ) scores were for vegetative variables ( $h_{m\text{\_}BS\;}^{2}=0.21,\;{\;h}_{m\text{\_}VB\;}^{2}=0.8$ ) and NP ( $h_{m_{NP}}^{2}=0.17$ ), and the lowest missing heritability values were for the yield component YLP ( $h_{m_{NP}}^{2}=0.05$ ) and NS ( $h_{m_{NP}}^{2}=0.02$ ).

### 2.5. A Total of 13 Customized SNP-Chips Captured Trait Variation Across Localities

**Figure 2:**
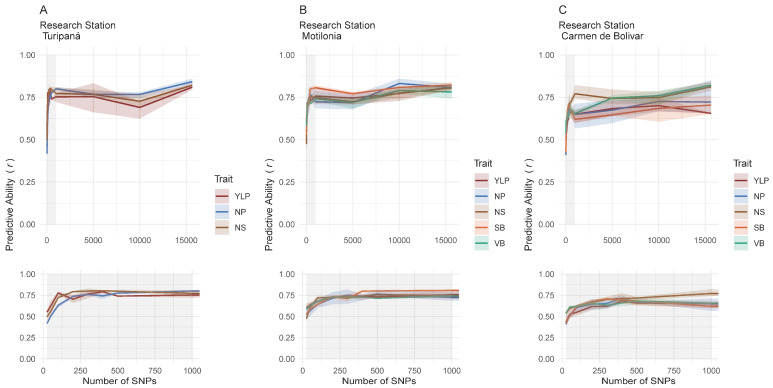
SNP marker saturation curves were iteratively reconstructed with the aim of finding the threshold that optimizes the number of SNPs while retaining high predictive ability using BayesC modeling for all yield components and biomass variables. The colored shadow around each tendency line is drawn according to the minimum and maximum values. The gray ribbon is a zoom-in, displayed at the bottom, of the curve between the 25 to 1000 SNP markers. Research stations: (**A**) Turipaná, (**B**) Motilonia, and (**C**) Carmen de Bolivar.

We iteratively retrieved all estimates of the modeling with the BayesC algorithm using marker datasets incrementally selected according to their *β* effects as follows: 25, 50, 100, 200, 300, 400, 500, 1000, 5000, 10,000, and 15,645 SNPs (Table S3). This way, we could plot the saturation curve (Figure 2) to determine a plateau that would allow for optimizing a threshold for SNP markers without risking the efficiency of the predictive ability. An initial plateau was found between 500 and 1000 SNP markers; therefore, a cut-off threshold of 500 was defined. This subsampling maintained high predictive ability for all traits, with a mean of 0.73 (±0.05) for YLP, 0.74 (±0.05) for NS, 0.71 (±0.05) for NP, 0.74 (±0.03) for SB, and 0.74 (±0.07) for VB. A subset of 500 SNP markers was adjusted for each trait and each locality, leading to a total of 13 customized SNP-chips.

**Figure 3:**
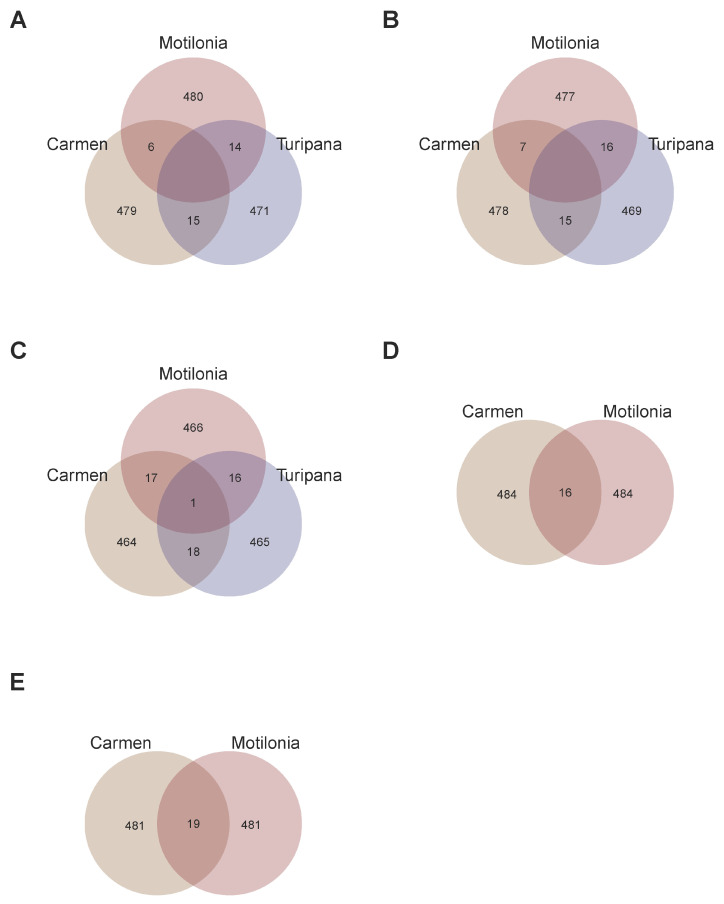
SNP sets aiming to optimize candidate SNP-chips for each trait in each locality. Venn diagram of the three SNP-chips in Motilonia, Turipaná, and Carmen de Bolivar for (**A**) yield per plant (YLP), (**B**) number of seeds (NS), and (**C**) number of pods (NP). Venn diagram of the two SNP-chips in Motilonia and Carmen de Bolivar for (**D**) seed biomass (SB), and (**E**) vegetative biomass (SB).

As a second step, we compared candidate SNP-chips per trait across localities to understand the interaction of the retrieved genomic architecture with the specific locality (Figure 3). All SNP-chips were at least 92.8% exclusive to each locality given a single trait. For the YLP variable, the SNP-chips only shared 5.8% of SNPs across localities (Figure 3A). For the NS trait, the SNP-chips only shared 6.2% of SNPs across localities (Figure 3B). Also, for the NS trait, the SNP-chips only shared 7.2% of SNPs across localities (Figure 3C). This limited overlap was also observed for the biomass variables; for example, for the SB trait, the SNP-chips only shared 3.2% of SNPs across localities (Figure 3D). In the same way, for the VB trait, the SNP-chips only shared 3.8% of SNPs across localities (Figure 3E).

**Figure 4:**
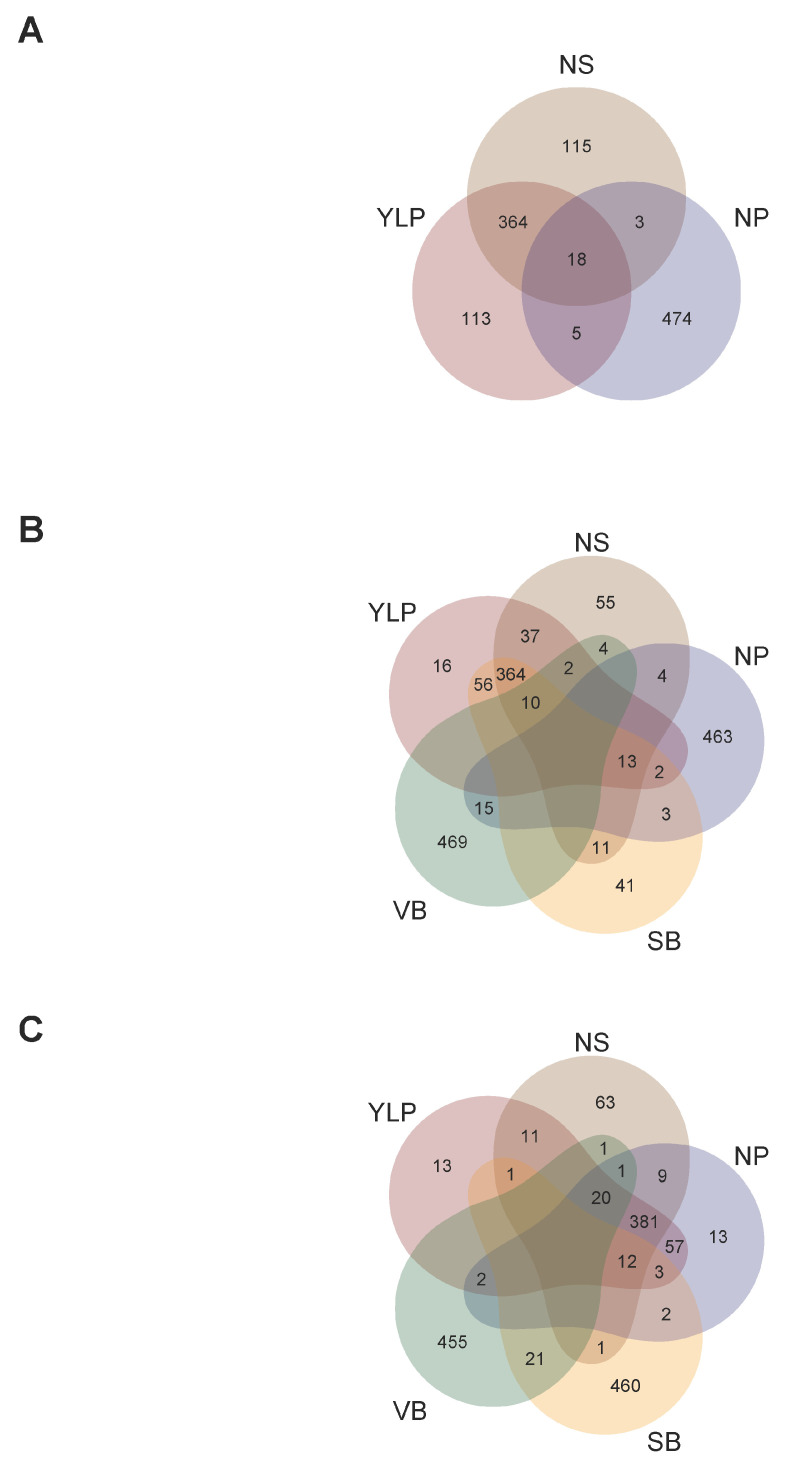
SNP sets aiming to optimize candidate SNP-chips for all traits in each locality. (**A**) Venn diagram of the three SNP-chips for YLP, NS, and NP in Turipaná. (**B**) Venn diagram of the five SNP-chips for YLP, NS, NP, SB, and VB in Carmen de Bolivar. (**C**) Venn diagram of the five SNP-chips for YLP, NS, NP, SB, and VB in Motilonia. Traits coded as: yield per plant (YLP), number of seeds per pod (NS), number of pods (NP), seed biomass as seed weight (SB), and vegetative biomass (VB).

As a third step, we compared candidate SNP-chips among traits at each locality to optimize targeted marker genotyping at single given localities (Figure 4). In this sense, a single SNP-chip for Turipaná required a total of 1092 unique markers because among the three SNP-chips (for the YLP, NS, and NP traits), 35.71% were shared by at least two traits (Figure 4A). Likewise, a single SNP-chip for Carmen de Bolivar required a total of 1565 unique markers because among the five SNP-chips (for the YLP, NS, NP, SB, and VB traits), 33.29% were shared by at least two traits (Figure 4B). A single SNP-chip for Motilonia required a total of 1526 unique markers because among the five SNP-chips (for the YLP, NS, NP, SB, and VB traits), 34.27% were shared by at least two traits (Figure 4C).

### 2.6. Recommendation Domains for Adaptation of Interspecific Genotypes

**Figure 5:**
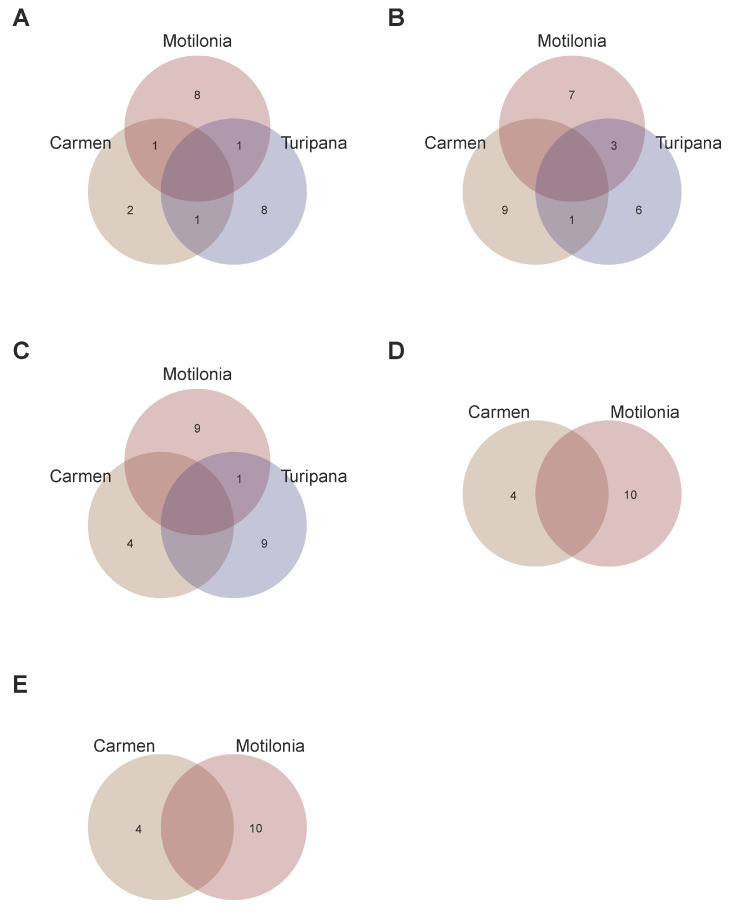
Genotype sets aiming to optimize candidate elite genotypes for each trait in each locality. The top 10 genotypes with the highest GEBV scores were considered for each trait in each locality. Venn diagram of the top genotypes in Motilonia, Turipaná, and Carmen de Bolivar for (**A**) yield per plant (YLP), (**B**) number of seeds (NS), (**C**) and number of pods (NP). Venn diagram of the top genotypes in Motilonia and Carmen de Bolivar for (**D**) seed (SB) and (**E**) vegetative (VB) biomass.

In order to recommend what genotypes to plant in which localities, we summarized genotype overlap using the top 10 thresholds according to their GEBVs (Figure 5). No single genotype was shared among the three retained localities for each trait. Therefore, a recommendation domain for narrow adaptation was more suitable given the current data. Three genotypes were recommended for the YLP trait due to their high GEBV in at least two of the three localities (G55 for Carmen de Bolivar and Motilonia, G54 for Carmen de Bolivar and Turipaná, and G20 for G54 for Motilonia and Turipaná, Figure 5A). Similarly, four genotypes were recommended for the NS trait due to their high GEBV in at least two of the three localities (G3 for Carmen de Bolivar and Turipaná and G8, G12, and G14 for Motilonia and Turipaná, Figure 5B). Finally, one genotype was recommended for the NP trait because of its high GEBV in at least two of the three localities (G70 for Motilonia and Turipaná, Figure 5C). On the other hand, it was not possible to identify superior genotypes for the SB and VB traits in any locality due to modest GEBV scores (Figure 5D,E).

**Figure 6:**
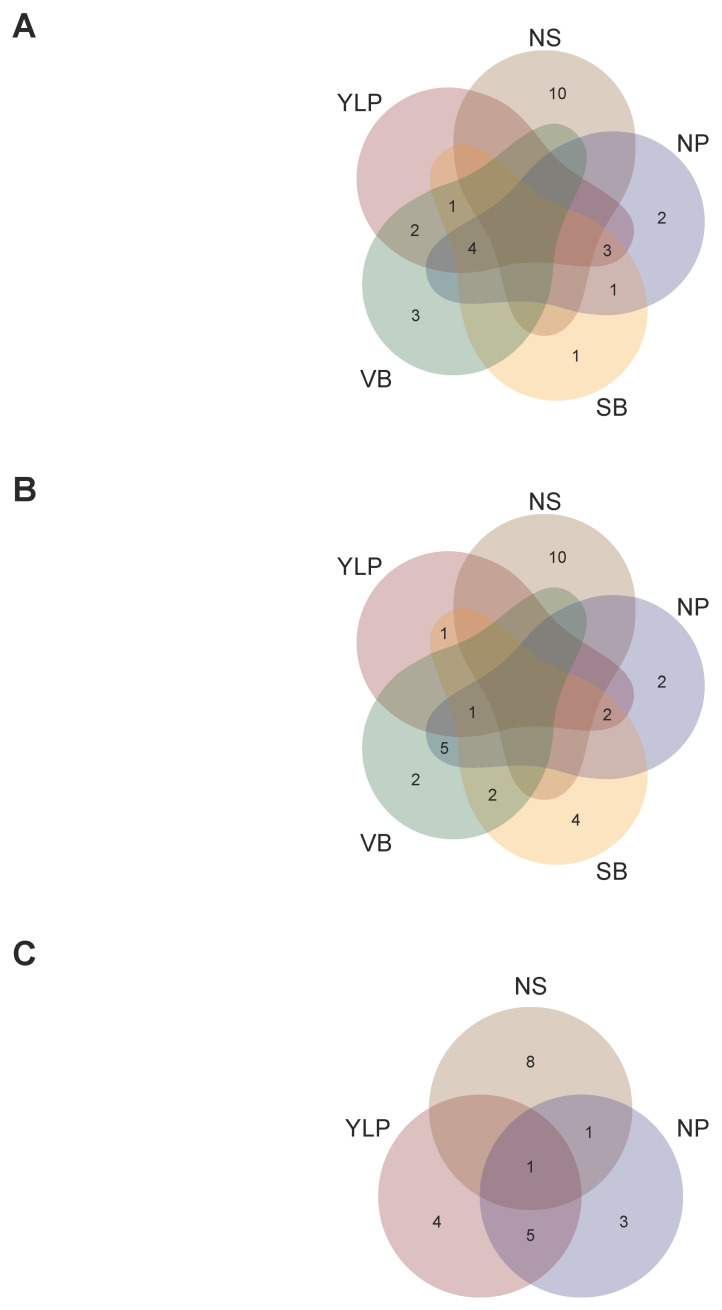
Genotype sets aiming to optimize candidate elite genotypes for all traits in each locality. (**A**) Venn diagram of the top genotypes for YLP, NS, NP, SB, and VB in Motilonia. (**B**) Venn diagram of the top genotypes for YLP, NS, NP, SB, and VB in Carmen de Bolivar. (**C**) Venn diagram of the top genotypes for YLP, NS, and NP in Turipaná.

Finally, we aimed to identify candidate superior genotypes for various traits at specific localities (i.e., multi-trait narrowly adapted lines). In this sense, G14 was the only genotype with a high GEBV value for all traits in Turipaná (Figure 6C). Similarly, G57 was the genotype encompassing elite variation for more traits (i.e., YLP, PN, SB, and VB) in Carmen de Bolivar (Figure 6B). Lastly, G20, G76, G77, and G78 simultaneously captured superior variation at several traits (i.e., YLP, PN, SB, and VB) in Motilonia (Figure 6A).

## 3. Discussion

In this study, we have implemented genomic prediction of performance in interspecific hybrids between *Phaseolus* species (i.e., common and Tepary beans) targeting the extreme environmental conditions of the Colombian Caribbean region. We have demonstrated that it is possible to use genomic prediction to predict yield components and biomass variables, specifically the yield per plant, number of pods, number of seeds, seed biomass, and vegetative biomass. Comparative analysis of multiple genomic prediction models indicates that the model family with the best predictive ability when using the complete set of markers was the Bayesian alphabet, with BayesC being the one with the best precision. Differences among model types vanish when relying only on the associated markers, yet their overall precision drops compared to the full marker set. The optimization of the most predictive SNP set reveals that 500 markers were enough to achieve maximum predictability. Using different training and validation datasets under an optimized marker scenario (500 SNPs), we also demonstrate promising genomic-enabled predictions for key traits such as the yield (*r*_YLP_ = 0.73), number of pods (*r*_NS_ = 0.71), number of seeds (*r*_NP_ = 0.74), seed biomass (*r*_SB_ = 0.74), and vegetative biomass (*r*_VB_ = 0.74). Based on the above model calibration and the obtained GEBV scores, we pinpoint superior genotypes per trait at each locality as a selection recommendation for future introgressive breeding cycles. We also encourage trait-specific SNP-chips for interspecific genotypes between common and Tepary beans and generalized chips for multiple localities. These models will guide further breeding among bean species targeting the Colombian Caribbean.

### 3.1. Genomic Prediction Assists Introgression Breeding

Authors such as Keller et al. [34] have explored the genomic prediction in common bean for agronomic traits, like 100-seed weight, days to flowering, days to physiological maturity, and seed yield, under humid and drought stress conditions in the Valle del Cauca province of Colombia. They obtained promising results with up to 0.6 predictive abilities for yield. However, genomic prediction of yield components and biomass variables have been little explored in hybrids between common beans and Tepary beans, which may harbor useful alleles for adaptation to regions with extreme weather when it comes to heat and drought stresses, such as the Colombian Caribbean [21,24].

Other authors have successfully explored genomic prediction for introgression breeding in other species such as maize [40], rice [41], sugar cane [42], and oil palm [43]. Inspired by them, we explored genomic prediction modeling for yield components and biomass variables in the framework of interspecific crosses for the case of common beans × Tepary beans. Our results achieved relatively high precision scores (above 0.71) in the Colombian Caribbean regions for all yield components and biomass variables, a promising outcome that will likely lead to the shortening of breeding cycles via indirect genomic selection. The predictive abilities reported here were higher than the scores for yield components obtained by Keller et al. [34] in Andean common beans and by Barili et al. [44] using Brazilian germplasm. Differences among predictive abilities of yield traits may be because we captured a greater number of SNPs (more predictor variables) than other studies, not to mention that we relied on interspecific crosses with germplasm of a species naturally adapted to dry and hot conditions (i.e., Tepary beans).

Improving traits such as grain yield or the number of pods in bean lines targeting a territory with high demand for dietary protein offers an opportunity to contribute to the food security of marginal communities. In addition, this study allowed us to explore an interspecific breeding strategy for a predominantly autogamous species, such as the common bean. After all, it is feasible to modernize strategies for current food security issues by relying on nature-based solutions and introgressive breeding.

### 3.2. Genomic Prediction Captures Missing Heritability and Locality-Dependent Effects

For all yield components and biomass variables, the genomic heritability scores were superior to the GWAS-based estimates and tended to increase from the humid locality of Turipaná to the most dried locality of Motilonia. A possible explanation is that because the crosses have been recurrent with common beans, there is a better pre-adaptation to more humid localities, and many alleles still need to be fixed in the population for drought stress in drier localities, such as Motilonia. Meanwhile, the genetic variance registered at each locality will likely tend to fall as the breeding cycles progress since the population will begin to fix adaptive alleles in each locality. Finally, the heritability scores that we reported were high, perhaps due to additive genetic differences between the interspecific populations [45]. These trends suggest promising efficiency of genomic selection due to high selection responses with moderate selection differentials.

### 3.3. Candidate Customized SNP-Chips for Genotype Ranking May Optimize Genomic Selection

The development and application of molecular markers in crop genetics have gained remarkable attention in the last three decades [46]. The tendency has recently culminated in abundant SNP markers based on next-generation sequencing technologies [47]. When extended to hybrid breeding, authors such as Ma et al. [48] and Yu et al. [49] in maize and Li et al. [50] in soybean have built specific SNP-chips for target traits and localities. Envisioning the same strategy, we made a specific SNP-chip for each trait and locality. It was even possible to obtain a general panel for multiple localities or various traits. The specific chip design that we suggest should be validated and confirmed in future breeding cycles, optimizing in this way sequencing efforts (time and cost), SNP calling reliability, bioinformatic processing, and downstream analytical steps.

### 3.4. Enhancing the Predictive Ability of GP for Interspecific Panels

The main goal of genomic prediction in hybrid breeding is to use the parents’ genotype to predict the hybrids’ performance, which would reduce the number of crosses to be tested in the field. Since there is no agreement as to which is the best model against an interspecific panel, it is still useful to perform comparative analyses, such as pilot screening, to choose the best model. For example, for general agronomic traits, the RKHS algorithm in sugarcane had better performance against seven other approaches [42], but for the same species, other authors reported better performance of the Bayesian alphabet against more than six models [51]. Similarly, BayesB and RKHS were the best against 15 approaches in rice [41]. In our case, BayesC exhibits the best performances throughout localities and traits.

On the other hand, authors such as Zhang et al. [36] or Spindel and McCouch [52] suggested the potential use of previous GWAS association studies to improve genomic prediction, an approach validated with real data by authors such as Sehgal et al. [53] and Shi et al. [54]. They demonstrated an optimization of up to 10% in the prediction ability by relying on prior genetic mapping inferences. We obtained results that differ from the above. On average, for all localities, a prediction ability of 0.61 (SD = 0.21) was obtained when using the associated markers derived from previous GWAS, which contrasts with an average predictive ability for all localities of 0.71 (SD = 0.14) when using all markers and a predictive ability of 0.57 (SD = 0.06) using an equivalent number of markers (i.e., 50) with the largest *β* effects by forward modeling with BayesC. This suggests that modeling genomic prediction with associated markers based on previous GWAS (47 SNPs) models is a more effective strategy than using approximately the same number of SNP markers ranked by their *β* effects in Bayesian regression (50 SNPs). However, possibly due to the highly polygenic nature of the yield components and biomass variables, using the full set of markers or the 500 *β*-optimized SNPs is far more effective for hybrid breeding.

### 3.5. Perspectives

This study offers a basis for selecting introgressed bean genotypes targeting extreme heat and drought conditions. SNP-chips will assist in implementing genomic prediction of superior interspecific common bean × Tepary bean genotypes in contrasting open-field localities of the Colombian Caribbean. Future studies aiming to advance complementary and recurrent backcrossing schemes must acknowledge that novel interspecific crossing schemes will benefit from using an optimized genomic prediction platform that relies on the *β* effects to minimize the number of markers needed to implement candidate customized SNP-chips in target traits and localities. We look forward to seeing more studies that follow these lines in the upcoming years. On the other hand, based on the infinitesimal additive predictive model [28], whole-genome resequencing may provide much more information for genomic prediction [55]. Still, we managed to obtain satisfying prediction abilities using marker depuration via GBS, likely due to the massive LD observed in the autogamous common and Tepary bean genomes [56]. In our particular case, GWAS approaches prior to GP modeling can be useful for SNP assays of low quantity (<50 markers), such as KASPar, which are already standardized for common beans [57]. Yet, SNP-chips able to rely on hundreds and thousands of SNP markers offer higher prediction after optimizing the full set according to their *β* effects.

Meanwhile, innovative genomic-assisted predictive methods have been developed under classic machine learning algorithms such as Random Forest [58], Support Vector Machine [59], boosting family [60], and deep learning [61]. Authors such as Azodi et al. [59] or Abdollahi-Arpanahi et al. [60] have extensively explored their efficiency, but further validation is required while containing the phantom of overfitting. Despite these developments, Bayesian regression approaches, such as the Bayesian alphabet, nonparametric regressions, like RKHS, and classical regression regularization approaches, like BRR and BL, still offer substantial precisions [62], as we have shown in this study.

Finally, the genotypes identified in this work as superior candidates for abiotic stress tolerance have the potential to leverage not only the following hybrid breeding cycles in the Caribbean region of Colombia but also improvement programs for abiotic stress tolerance in Africa and South and Central America, where similar heat and water scarcity regimens are already observed. Future studies should aim to validate the trait- and locality-specific SNP-chips proposed in this study across more advanced breeding cycles, novel localities, and contrasting environments. Ultimately, this work sets a pivotal step in climate-resilient breeding for tropical legumes, potentially accelerating the deployment of heat-resilient beans where they are most needed. Data-driven genomic forecasting is then essential to meet future food demands under a changing climate [63].

## 4. Materials and Methods

### 4.1. Plant Material and Multi-Locality Field Trials

The panel of 87 genotypes utilized in this study was composed of 67 interspecific lines between common beans (*P. vulgaris*) and Tepary beans (*P. acutifolius*) and 19 advanced genotypes bred in high temperature and drought conditions by the bean program of the Alliance Bioversity–CIAT (International Center for Tropical Agriculture) and transferred to AGROSAVIA after material transfer agreement (MTA) subscription. Also, we used the genotype G40001 (*P. acutifolius*) as a control. The interspecific lines were obtained from the third generation onwards (detailed pedigree in Table S1). This panel of genotypes was evaluated for the first time at four localities in the humid and dry Colombian Caribbean sub-regions [21,24] during the crop cycle of July–October 2020. However, to better control the coefficient of variation, this study focused on three of them.

The localities in the humid and dry Colombian Caribbean sub-regions corresponded to the following AGROSAVIA’s research stations: Motilonia ([10°00′01.2″ N, 73°15′22.4″ W] in the municipality of Codazzi in the province of Cesar), Carmen de Bolívar ([9°42′50.8″ N, 75°06′26.9″ W] in the municipality of Carmen de Bolívar in the province of Bolívar), and Turipaná ([8°50′27.47″ N, 75°48′27.56″ W] in the municipality of Cereté in the province of Córdoba). The research station Turipaná (tropical plains at less than 20 m a.s.l.) was representative of the humid Caribbean sub-region, while the research stations Motilonia and Carmen de Bolivar (mountainous and foothills, both at more than 100 m a.s.l.) belonged to the dry Caribbean sub-region. Average minimum and maximum temperatures oscillated from 23 °C to 25 °C (average of 23.7 °C) and from 33 °C to 36.3 °C (average of 33 °C) for the dry and humid Caribbean sub-regions, respectively. Relative humidity and precipitation during the rainy season varied from 70% to 80% (average of 80%) and from 482 mm to 700 mm (average of 591 mm) for the dry and humid Caribbean sub-regions [21].

### 4.2. Experimental Design and Phenotypic Segregation Across Localities

Genotypes were planted following a completely randomized block design (CRBD) with three repetitions at each locality. Standard traits [21,64] in common beans were measured at the end of the cycle at each locality: YLP, yield per plant (g/plant); NP, number of pods per plant; NS, average number of seeds per pod; SB, seed biomass as seed weight (g); and VB, vegetative biomass (g). Raw data is available in [21,24].

The phenotypic descriptive analyses in López-hernández et al. [24] suggested among-locality trait segregation for most of the studied interspecific genotypes. This phenotypic segregation was recurrent in a second field trial carried out in 2022-I using the same panel of interspecific genotypes. With the goal of weighing intra-genotype variability across localities for each yield trait, López-hernández et al. [24] proposed an index that ponders the variability in each trait as the ratio of the mean of each genotype and its variance. Thus, high index values indicated genotypes with high performance and uniformity. Also, we computed a correlation matrix between the yield components (YLP, NS, NP) and biomass variables (SB and VB) using parametric (Pearson’s correlation coefficient) and nonparametric (Spearman’s rank correlation coefficient) approaches corrected by the Bonferroni test through the function *ggcorrmat* in the R-package *ggstatsplot*.

### 4.3. Genotyping by Sequencing and SNP Calling

Genomic data was obtained by means of genotyping by sequencing (GBS) [65]. The DNA extraction was carried out using AGROSAVIA’s in-house protocol from leaf tissue sampled 40 days after germination. The enzymatic digestion was carried out using the cutting enzyme *Apek1*, standardized for common beans as part of previous studies [66,67]. The genotyping of the interspecific panel is further described in [24].

DNA sequences were obtained by the Illumina 2500 Hiseq sequencer (Macrogen, Seoul, Republic of Korea) in a single direction (single end). After the sequencing quality analysis reported in [24], an automatized SNP calling script was constructed using the function *HaplotypeCaller* of the protocol GATK4 [68] with the alignment algorithm BWA [69] to identify allelic polymorphisms. We used the second annotated assembly version of the reference genome for *P. vulgaris*, as downloaded from the *Phytozome* platform with an overall extension of ~600 Mb and a read depth of ~83.2× (*P. vulgaris* v2.1, DOE-JGI and USDA-NIFA, http://phytozome.jgi.doe.gov/, accessed on 19 June 2025). Mapping statistics were performed by the function *flagstat* in Samtools v.1.9 software [70] from the platform of the Galaxy project 2.0.3 [71]. We filtered the SNP matrix in the software Tassel 5.2.78 [72] using a maximum percentage of missing data of 20% by loci and by sample, a minimum depth of 3×, and a minimum allele frequency (*maf*) of 5%.

To improve the accuracy of the prediction models, we imputed missing genotypes using LinkImpute for non-model organisms [73] as follows: 10 nearest neighbors, 30 sites in high-linkage disequilibrium (LD), and ${\;10\times 10}^{6}$ as the maximum distance between sites to compute LD. The SNP calling process of the interspecific panel led to 15,645 SNPs, as detailed in [24] (GitHub repository: https://github.com/FelipeLopez2019/SNP-calling-of-KOLFACI-project/blob/main/Kolfaci_Colombia_v4.sh, accessed on 19 June 2025).

As a last validation step on the resultant SNP matrix, Bayesian phylogenetic inference was performed using all 15,645 SNPs, with *P. acutifolius* (accession G87) as an outgroup. The evolutionary model applied was the general time reversible (GTR) model. Phylogenetic reconstruction was conducted using the MrBayes v3.2.6 plugin within Geneious v9.1.8. The analysis employed a Markov Chain Monte Carlo (MCMC) algorithm with four chains, each running for 100,000 generations, a chain temperature of 0.2, and a sampling frequency of every 50 generations to optimize computational memory usage. A burn-in of 1000 generations was applied to discard the initial phase of the Markov Chains to reduce the influence of early noise in the model. The Bayesian phylogenetic analysis recovered three major clades corresponding to the Mesoamerican and Andean gene pools of *P. vulgaris* and *P. acutifolius* (Figure S1), matching the results previously reported by López-Hernández et al. [24] through ancestry analysis and unsupervised learning.

### 4.4. Genomic Datasets from GBS and GWAS

Complex quantitative traits usually display a polygenetic basis with minor loci effects, following the infinitesimal additive genetic model [28]. Given this definition, previous reports in common beans that have characterized the genomic architecture of yield components [24,55] and biomass variables [24,74] are in agreement with the polygenetic hypothesis. Given the number of markers required to reconstruct the genetic bases of polygenic traits, and with the aim to optimize the genotyping effort, authors such as Keller et al. [34] and Arenas et al. [29] have explored the reduction in the SNP panel while maintaining the accuracy of genomic prediction. Specifically, the optimization of SNP datasets has been performed by controlling LD redundancy among markers, as in Keller et al. [34] for beans, Arenas et al. [29] in pine trees, and Tan et al. [35] for the eucalyptus breeding program in Brazil. Additionally, other authors have explored using associated markers from previous GWAS to weight the marker input in GP algorithms, retaining similar accuracies compared to the full marker set [36,37]. To optimize the genomic dataset for genomic prediction, we considered two SNP matrices, one comprising all markers from the original GBS screening and the other SNP matrix only containing associated markers outputted from previous GWAS models for the same traits and populations [24]. Therefore, the former raw GBS matrix comprised all 15,645 SNP markers, while the latter GWAS-filtered matrix contained 43 associated markers.

### 4.5. Genomic Prediction Analyses

Genomic prediction provides a more complete representation of a quantitative polygenic trait than traditional GWAS-based genetic mapping because the latter performs poorly in capturing small effects or rare variants [75]. GP utilizes phenotypic data [30] to calibrate marker-based additive infinitesimal predictive models [27,28,29]. The statistical challenge is then to estimate the effects of individual SNPs in a case where the number of individuals being trained is much smaller than the large number of SNPs [76]. Therefore, several Bayesian regression approaches have been explored such as BayesA [28], BayesB [28], BayesC [76], LASSO [77], and Ridge [78]. Other nonparametric genomic regressions have also been tested, such as the algorithm based on Reproducing Kernel Hilbert Spaces (RKHS) methods [27,33,79]. All models were run as in https://github.com/FelipeLopez2019/Genomic_prediction_Lopez-Hernandez-et-al-2023 (accessed on 19 June 2025).

Specifically, we compared six different methods for the genomic prediction of yield performance and biomass traits across interspecific bean lines. Specifically, the trait of interest y was modeled with a linear combination of m SNPs, expressed as follows:

(1) $$y=\mu 1_{n}+X\beta +\varepsilon$$

where y is an *n*-vector of phenotypes measured in n individuals; X is an n×m matrix of genotypes screened at m SNPs; μ is the intercept (i.e., population average); β is a m -vector of SNP effects to be estimated; and ε is an ***n***-vector of normally distributed residuals, $\varepsilon \;\sim \;N\left(0,\sigma_{e\;}^{2}\;I_{n\times n}\right)$ .

The usual method of model estimation, the method of least squares, produces unbiased estimators so that under many predictors (i.e., SNP markers), the variance of the estimators increases. To improve the predictions (reduce the variance of the estimators), penalization (or regularization) methods have been proposed as Least Absolute Shrinkage and Selection Operator (LASSO) and ridge regularization algorithms. These approaches force the model coefficients to zero, thus minimizing the risk of overfitting, controlling variance, attenuating the correlation effect between predictors, and reducing the influence of less relevant predictors on the model. In a similar way, two Bayesian hierarchical methods, BayesA and BayesB [28], have shown through real data and simulations that the accuracies of GEBVs are higher with Bayesian methods than with least squares or Ridge regression [28,80,81]. BayesC [76] is an improvement of BayesA and BayesB that disregards prior SNP effects. In BayesA and BayesB, the probability π that a single SNP has zero effect is treated as known, so in BayesA, π=0 (all SNPs have non-zero effect), and in BayesB, π>0 (assumes that many SNPs have a zero effect) [76]. BayesC treats π as an unknown, and so it is estimated from the same data. Bayesian inference was used to estimate the GP models’ hyperparameters [30,31].

Following de los Campos et al. and Ferrão et al. [30,82], the posterior distribution of the model parameters $\mu ,\;\beta ,\sigma^{2}$ given the hyperparameters ω can be expressed as follows:

(2) $$p\left(\;\mu ,\;\beta ,\;\sigma^{2}|y,\omega \right)\;\alpha \;p\;(y\left|\mu ,\;\beta ,\;\sigma^{2}\right)\;p(\mu ,\;\beta ,\sigma^{2}|\omega )$$

where $p\left(\mu ,\beta ,\sigma^{2}|y,\omega \right)$ is the posterior probability density of the parameters $\mu ,\beta ,\sigma^{2}$ given the data vector **y** and the hyperparameters ω . The regression likelihood from Equation 1 is the term $p(y\left|\mu ,\beta ,\sigma^{2}\right)$ , while $p(\mu ,\beta ,\sigma^{2}|\omega )$ is the prior density distribution of the model parameters. All Bayesian algorithms primarily differ in how the priors are assigned to the regression coefficients and other model hypermeters [83].

On the other hand, we explored the semiparametric approach RKHS [27] to explore alternative non-parametric distributions that may be more suited for the studied population [29]. Without making strong a priori assumptions on the distribution of marker effects ( β ), this method allows for inferring individual functions for specific SNP markers. As an alternative, the SNP marker function predicts genomic-enhanced genotypic values if the Gaussian Kernel encodes additive effects, which depends on a bandwidth parameter ( h ) [84]. All methods, BayesA, BayesB, BayesC, Bayesian LASSO, Bayesian Ridge, and RKHS, were implemented in the BGLR R-package [85] with 10,000 Monte Carlo Markov Chains (MCMCs) and a burn-in of 1000 steps. We adopted the default hyperparameters with the original configuration (prior density) described in Pérez and de los Campos [85].

### 4.6. Predictive Ability and Genomic Heritability

All six implemented methods were evaluated based on their predictive ability ( $r_{y}$ ) estimated for each yield component and biomass variable and per genomic dataset (full SNP dataset and only associated markers). The predictive ability was computed as the Pearson correlation coefficient between the vector of the observed phenotypic variable y and the GEBV vector [29]. 

After that, we estimated the narrow sense heritability $\left(h^{2}\right)$ using the genomic heritability $\left(h_{g}^{2}\right)$ as in de los Campos et al. [86], following Equation (3) as follows:

(3) $$h_{g}^{2}=\frac{\sigma_{a}^{2}}{\sigma_{a}^{2}+\sigma_{e}^{2}}$$

where $\sigma_{a}^{2}$ is the additive variance and $\sigma_{e}^{2}$ is the residual variance. Both variances were calculated for each trait (yield components and biomass variables) and set of SNPs (full GBS SNP matrix and GWAS-filtered SNP matrix) [29,87,88]. Also, we explore the missing heritability ( $h_{m}^{2}$ ) between the genomic heritability from the full SNP set ( $h_{G}^{2}$ ) and the one obtained from only the associated markers $\left(h_{GWAS}^{2}\right)$ . In this sense, the missing heritability between the associated markers and the total markers was calculated as follows:

(4) $$h_{m}^{2}=\frac{{h_{G}^{2}-h}_{GWAS}^{2}}{h_{G}^{2}}$$

On the other hand, assuming statistical independence between observations and the Gaussian distribution, we obtained the mean squared errors (MSEs) as follows:

(5) $$MSE\left(\bar{X}\right)=E\left({\left(\bar{X}-\mu \right)}^{2}\right)={\left(\frac{\sigma }{\sqrt{n}}\right)}^{2}$$

To break any overfitting due to the data structure, we performed a cross-validation (CV) by implementing a random subsampling partitioning of the data in five folds. For each replicate (i.e., fold), the values $r_{y}$ , $h_{g}^{2}$ , and ***MSE*** were retained. Finally, we evaluated significant differences for $r_{y}$ following Tan et al. [35] and Arenas et al. [29] among GP approaches (BayesA, BayesB, BayesC, BRR, BL, and RHKS) across the type of trait (yield components and biomass variables) and set of SNPs (full GBS matrix and GWAS-filtered matrix) through a one-way ANOVA and Kruskal–Wallis test using the *ggbetweenstats* function in the R-Package *ggstatsplot* [39]. Due to different sample sizes per locality, a Dunn ad hoc test was implemented using Bonferroni’s *p*-value adjustment method, which was also performed in the *ggbetweenstats* function in the R-Package *ggstatsplot* [39].

After screening all models, we selected the BayesC approach to explore the data behavior regarding prediction ability and heritability across all three localities and the five yield components and biomass variables in each SNP set. We used the one-way ANOVA with the Mann–Whitney U test to detect significant differences between the two SNP sets (i.e., all markers and only GWAS-inferred associated markers) with *ggstatsplot* [39], too.

### 4.7. Candidate Markers for Customized SNP-Chips per Trait and Locality

Since BayesC was the GP approach with the best performance, we sorted all markers (15,645 SNPs) according to the *β* effects of the Bayesian regression model for each yield component and biomass variable (YLP, NS, NP, SB, and VB) across the top localities (Carmen de Bolivar, Motilonia, and Turipaná). Thus, we constructed 10 new SNP sets with the most predictive 25, 50, 100, 200, 300, 400, 500, 1000, 5000, and 10,000 SNP markers according to their *β* effects. Model optimization used Monte Carlo Markov Chains (MCMCs) with 10,000 iterations and 1000 burn-in steps with five-fold random cross-validation (CV) subsampling. Each model was run using three different seeds (“0000”, “1234”, “2023”) to break stochastic biases. We recorded the prediction ability values from all models and plotted the saturation curve to find the threshold that optimized the number of SNPs capable of retaining predictive abilities as the entire SNP dataset.

Meanwhile, with the aim to report how many molecular markers were in the same SNP-chip of each yield component and biomass variables, we constructed Venn diagrams across traits at each locality. Finally, we compared the prediction ability between the optimized SNP-chip per locality and the SNP-chip optimized according to previous GWAS models (i.e., only containing associated markers).

### 4.8. Top Genotypes per Locality

The genomic estimated breeding values (GEBVs) were retrieved from the best-performing model for each yield component and biomass variable across localities. Bar graphs were drawn to determine the inflection point in the GEAV scores, indicating that 10 genotypes served as a general threshold to determine the top genotypes for each model. Finally, the lists of elite genotypes were summarized using Venn diagrams to suggest broad and narrow adaptation recommendation domains across localities.

## 5. Conclusions

The current work offers a robust framework for accelerating introgression breeding in common beans through the integration of interspecific hybridization and genomic prediction under extreme climatic conditions in terms of heat and drought, such as those found on the Caribbean coast of Colombia. This study in turn highlights an efficient strategy to capture both polygenic adaptation and locality-dependent effects, demonstrating high predictive abilities for yield components and biomass traits, using Bayesian regression (i.e., particularly BayesC) and optimizing SNP sets to 500 markers. The superior performance of full marker genomic prediction over a priori GWAS-informed or reduced-marker models underscores the importance of accounting for missing heritability in complex traits through low-effect and low-frequency molecular markers. The suggested trait- and locality-specific SNP-chips offer a practical tool for deploying genomic selection across diverse breeding scenarios, not only in the Colombian Caribbean but also in regions facing comparable abiotic stresses. This framework set the stage for more targeted, resource-efficient, rapid, and precise breeding cycles [89] while contributing to broader goals of food security by enhancing the adaptability and productivity of local varieties of high-protein and dietary fiber legume crops in the face of climate change [90].

## Figures and Tables

**Table 1:** Summary of genomic heritability (***h*^2^_g_**), prediction ability (***r_y_***), and mean squared errors (MSEs) from BayesC modeling for each yield component and biomass variables across localities (research stations) using all markers and only a priori GWAS-associated markers. The raw data is available in Table S2. Traits are coded as follows: yield per plant (YLP), number of seeds per pod (NS), number of pods (NP), seed biomass as seed weight (SB), and vegetative biomass (VB).

Variable	Locality	SNP Dataset	Best-Performing Model	Prediction Ability *r_y_*	Genomic Heritability *h*^2^_g_	MSE
YLP	Carmen de Bolivar	All markers	BayesC	0.83 ± 0.17	0.487 ± 0.015	0.038
YLP	Carmen de Bolivar	Associated markers	BayesC	0.67 ± 0.18	0.378 ± 0.004	0.055
YLP	Motilonia	All markers	BayesC	0.81 ± 0.10	0.776 ± 0.009	0.010
YLP	Motilonia	Associated markers	BayesC	0.80 ± 0.12	0.719 ± 0.003	0.010
YLP	Turipaná	All markers	BayesC	0.79 ± 0.10	0.289 ± 0.016	0.093
YLP	Turipaná	Associated markers	BayesC	0.53 ± 0.20	0.175 ± 0.003	0.169
NP	Carmen de Bolivar	All markers	BayesA	0.47 ± 0.12	0.466 ± 0.008	0.093
NP	Carmen de Bolivar	Associated markers	BayesC	0.49 ± 0.12	0.251 ± 0.003	0.093
NP	Motilonia	All markers	BayesC	0.82 ± 0.10	0.760 ±0.009	0.015
NP	Motilonia	Associated markers	BayesC	0.77 ± 0.11	0.601 ± 0.004	0.018
NP	Turipaná	All markers	BayesC	0.84 ± 0.13	0.597 ± 0.016	0.033
NP	Turipaná	Associated markers	BayesC	0.46 ± 0.18	0.345 ± 0.003	0.067
NS	Carmen de Bolivar	All markers	BayesC	0.83 ± 0.18	0.402 ± 0.015	0.037
NS	Carmen de Bolivar	Associated markers	BayesC	0.70 ± 0.18	0.340 ± 0.004	0.053
NS	Motilonia	All markers	BayesC	0.81 ± 0.09	0.670 ± 0.012	0.010
NS	Motilonia	Associated markers	BayesC	0.79 ± 0.12	0.727 ± 0.005	0.010
NS	Turipaná	All markers	BayesC	0.85 ± 0.08	0.224 ± 0.011	0.097
NS	Turipaná	Associated markers	BayesC	0.48 ± 0.20	0.181 ± 0.002	0.168
SB	Carmen de Bolivar	All markers	BayesC	0.84 ± 0.17	0.680 ± 0.013	0.030
SB	Carmen de Bolivar	Associated markers	BayesC	0.71 ± 0.18	0.388 ± 0.004	0.039
SB	Motilonia	All markers	BayesC	0.83 ± 0.10	0.744 ± 0.007	0.018
SB	Motilonia	Associated markers	BayesC	0.81 ± 0.12	0.513 ± 0.006	0.021
VB	Carmen de Bolivar	All markers	BayesC	0.75 ± 0.10	0.455 ± 0.012	0.018
VB	Carmen de Bolivar	Associated markers	BayesC	0.47 ± 0.20	0.256 ± 0.002	0.256
VB	Motilonia	All markers	BayesC	0.83 ± 0.08	0.874 ± 0.006	0.018
VB	Motilonia	Associated markers	BayesC	0.83 ± 0.11	0.793 ± 0.004	0.019

## Data Availability

Data is contained within the article and Supplementary Materials. The pipeline code is available in the GitHub repository as detailed in the Section 4.

## Supplementary Materials

The following supporting information can be downloaded at https://www.mdpi.com/article/10.3390/ijms26157370/s1.
