# Supplementary material for: Genomic Prediction of Adaptation in Common Bean (Phaseolus vulgaris L.) × Tepary Bean (P. acutifolius A. Gray) Hybrids
Source: Int J Mol Sci. 2025 Jul 30;26(15):7370. doi: 10.3390/ijms26157370 (PMC12347012; doi:10.3390/ijms26157370)
Supplement: Supplementary file 1 [file ijms-26-07370-s001.zip › FigureS3.pdf]

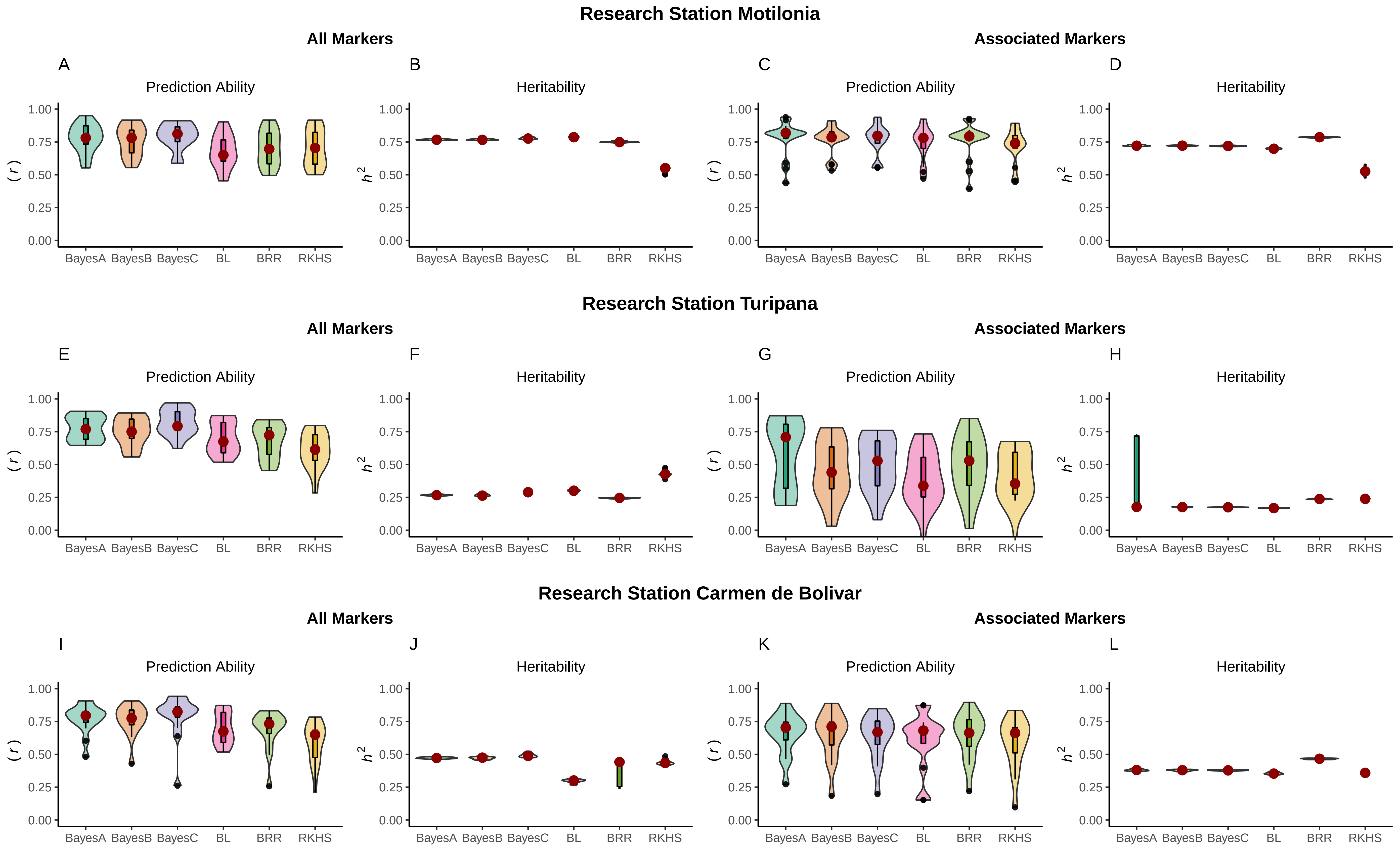
Research Station Motilonia
All Markers
Associated Markers
A
B
C
D
Prediction Ability
Heritability
(r)
h2
1.00
0.75
0.50
0.25
0.00
BayesA
BayesB
BayesC
BL
BRR
RKHS
Research Station Turipana
E
F
G
H
Research Station Carmen de Bolivar
I
J
K
L
