# Supplementary material for: Genomic Prediction of Adaptation in Common Bean (Phaseolus vulgaris L.) × Tepary Bean (P. acutifolius A. Gray) Hybrids
Source: Int J Mol Sci. 2025 Jul 30;26(15):7370. doi: 10.3390/ijms26157370 (PMC12347012; doi:10.3390/ijms26157370)
Supplement: Supplementary file 1 [file ijms-26-07370-s001.zip › supplementary materials, tables and figures captions.pdf]

Supplementary Materials—Table and Figure Captions

Table S1. Genealogy of 87 genotypes, composed of 67 inter-specific lines between common bean (*P. vulgaris* L.) and Tepary bean (*P. acutifolius*), and 19 advanced genotypes bred to high temperature and drought conditions by the bean program of the Alliance Bioversity–CIAT. The genotype G40001 (*P. acutifolius*) was used as control. The panel of genotypes was evaluated for the first time at four localities in the humid and dry Colombian Caribbean sub-regions [1].

Table S2. Raw outputs of genomic heritability, prediction ability and mean square errors from Bayesian regression modeling (BayesA, BayesB, BayesC, BL, BRR, and RKHS) for each yield component and biomass variable across localities (research stations) in coastal Colombia using all markers and only GWAS-associated markers. For the Bayesian modeling, we carried out Monte Carlo Markov Chain (MCMC) with 10,000-time iterations and 1000 burn-in steps in the BGLR R-package [2]. We adopted the default hyperparameters with the original configuration (prior density) described in [2].

Table S3. Raw outputs of prediction ability from BayesC modeling for each yield component and biomass variables across localities (research stations) in coastal Colombia using incremental sets of markers ranked according to their β regression coefficients at cuts of 25, 50, 100, 200, 300, 400, 500, 1000, 5000 and 10,000 SNPs.

Figure S1. Bayesian phylogenetic analysis based on 15,645 SNP variants using MrBayes 3.2.6.

Figure S2. Correlation matrix between yield components (YLP, NS, NP) and biomass variables (SB and VB) using parametric (Pearson's correlation coefficient) and nonparametric (Spearman's rank correlation coefficient) approaches corrected using Bonferroni's multiple comparisons by means of the function *ggcorrmat* in the R-package *ggstatsplot*. **A.** Global correlation matrix by parametric approach. **B.** Global correlation matrix by nonparametric approach. **C.** Correlation matrices in each research station by parametric approach. **D.** Correlation matrices in each research station by nonparametric approach.

Figure S3. Prediction ability and heritability of GP modeling by BayesC for the yield per plant (YLP) trait using all markers and only a priori GWAS-associated markers across three research stations. **A.** Prediction ability using all markers in Motilonia. **B.** Heritability using all markers in Motilonia. **C.** Prediction ability using associated markers in Motilonia. **D.** Heritability using associated markers in Motilonia. **E.** Prediction ability using all markers in Turipaná. **F.** Heritability using all markers in Turipaná. **G.** Prediction ability using associated markers in Turipaná. **H.** Heritability using associated markers in Turipaná. **I.** Prediction ability using all markers in Carmen de Bolivar **J.** Heritability using all markers in Carmen de Bolivar. **K.** Prediction ability using associated markers in Carmen de Bolivar. **L.** Heritability using associated markers in Carmen de Bolivar.

Figure S4. Prediction ability and heritability of GP modeling by BayesC for the number of seeds per pod (NS) trait using all markers and only a priori GWAS-associated markers across three research stations. **A.** Prediction ability using all markers in Motilonia. **B.** Heritability using all markers in Motilonia. **C.** Prediction ability using associated markers in Motilonia. **D.** Heritability using associated markers in Motilonia. **E.** Prediction ability using all markers in Turipaná. **F.** Heritability using all markers in Turipaná. **G.** Prediction ability using associated markers in Turipaná. **H.** Heritability using associated markers in Turipaná. **I.** Prediction ability using all markers in Carmen de Bolivar **J.** Heritability using all markers in Carmen de Bolivar. **K.** Prediction ability using associated markers in Carmen de Bolivar. **L.** Heritability using associated markers in Carmen de Bolivar.

Figure S5. Prediction ability and heritability of GP modeling by BayesC for the number of pods (NP) trait using all markers and only a priori GWAS-associated markers across three research stations. **A.** Prediction ability using all markers in Motilonia. **B.** Heritability using all markers in Motilonia. **C.** Prediction ability using associated markers in Motilonia. **D.** Heritability using associated markers in Motilonia. **E.** Prediction ability using all markers in Turipaná. **F.** Heritability using all markers in Turipaná. **G.** Prediction ability using associated markers in Turipaná. **H.** Heritability using associated markers in Turipaná. **I.** Prediction ability using all markers in Carmen de Bolivar **J.** Heritability using all markers in Carmen de Bolivar. **K.** Prediction ability using associated markers in Carmen de Bolivar. **L.** Heritability using associated markers in Carmen de Bolivar.

Figure S6. Prediction ability and heritability of GP modeling by BayesC for the seed biomass (SB) trait measured as seed weight using all markers and only a priori GWAS-associated markers across three Research Stations. **A.** Prediction ability using all markers in Motilonia. **B.** Heritability using all markers in Motilonia. **C.** Prediction ability using associated markers in Motilonia. **D.** Heritability using associated markers in Motilonia. **E.** Prediction ability using all markers in Carmen de Bolivar **F.** Heritability using all markers in Carmen de Bolivar. **G.** Prediction ability using associated markers in Carmen de Bolivar. **H.** Heritability using associated markers in Carmen de Bolivar.

Figure S7. Prediction ability and heritability of GP modeling by BayesC for the vegetative biomass (VB) trait using all markers and only a priori GWAS-associated markers across three Research Stations. **A.** Prediction ability using all markers in Motilonia. **B.** Heritability using all markers in Motilonia. **C.** Prediction ability using associated markers in Motilonia. **D.** Heritability using associated markers in Motilonia. **E.** Prediction ability using all markers in Carmen de Bolivar **F.** Heritability using all markers in Carmen de Bolivar. **G.** Prediction ability using associated markers in Carmen de Bolivar. **H.** Heritability using associated markers in Carmen de Bolivar.

Figure S8. Mean squared error (MSE) in the training partition and MSE in the testing partition of GP modeling by BayesC for YLP using all markers and only a priori GWAS-associated markers across three research stations. **A.** MSE in the training partition using all markers in Motilonia. **B.** MSE in the testing partition using all markers in Motilonia. **C.** MSE in the training partition using associated markers in Motilonia. **D.** MSE in the testing partition using associated markers in Motilonia. **E.** MSE in the training partition using all markers in Turipaná. **F.** MSE in the testing partition using all markers in Turipaná. **G.** MSE in the training partition using associated markers in Turipaná. **H.** MSE in the testing partition using associated markers in Turipaná. **I.** MSE in the training partition using all markers in Carmen de Bolivar **J.** MSE in the testing partition using all markers in Carmen de Bolivar. **K.** MSE in the training partition using associated markers in Carmen de Bolivar. **L.** MSE in the testing partition using associated markers in Carmen de Bolivar.

Figure S9. MSE in the training partition and MSE in the testing partition of GP modeling by BayesC for the NS trait using all markers and only a priori GWAS-associated markers across the three Research Stations. **A.** MSE in the training partition using all markers in Motilonia. **B.** MSE in the testing partition using all markers in Motilonia. **C.** MSE in the training partition using associated markers in Motilonia. **D.** MSE in the testing partition using associated markers in Motilonia. **E.** MSE in the training partition using all markers in Turipaná. **F.** MSE in the testing partition using all markers in Turipaná. **G.** MSE in the training partition using associated markers in Turipaná. **H.** MSE in the testing partition using associated markers in Turipaná. **I.** MSE in the training partition using all markers in Carmen de Bolivar **J.** MSE in the testing partition using all markers in Carmen de Bolivar. **K.** MSE in the training partition using associated markers in Carmen de Bolivar. **L.** MSE in the testing partition using associated markers in Carmen de Bolivar.

Figure S10. MSE in the training partition and MSE in the testing partition of GP modeling by BayesC for the NP trait using all markers and only a priori GWAS-associated markers across the three

Research Stations. **A.** MSE in the training partition using all markers in Motilonia. **B.** MSE in the testing partition using all markers in Motilonia. **C.** MSE in the training partition using associated markers in Motilonia. **D.** MSE in the testing partition using associated markers in Motilonia. **E.** MSE in the training partition using all markers in Turipaná. **F.** MSE in the testing partition using all markers in Turipaná. **G.** MSE in the training partition using associated markers in Turipaná. **H.** MSE in the testing partition using associated markers in Turipaná. **I.** MSE in the training partition using all markers in Carmen de Bolivar **J.** MSE in the testing partition using all markers in Carmen de Bolivar. **K.** MSE in the training partition using associated markers in Carmen de Bolivar. **L.** MSE in the testing partition using associated markers in Carmen de Bolivar.

Figure S11. MSE in the training partition and MSE in the testing partition of GP modeling by BayesC for the SB trait using all markers and only a priori GWAS-associated markers across the three Research Stations. **A.** MSE in the training partition using all markers in Motilonia. **B.** MSE in the testing partition using all markers in Motilonia. **C.** MSE in the training partition using associated markers in Motilonia. **D.** MSE in the testing partition using associated markers in Motilonia. **E.** MSE in the training partition using all markers in Carmen de Bolivar **F.** MSE in the testing partition using all markers in Carmen de Bolivar. **G.** MSE in the training partition using associated markers in Carmen de Bolivar. **H.** MSE in the testing partition using associated markers in Carmen de Bolivar.

Figure S12. MSE in the training partition and MSE in the testing partition of GP modeling by BayesC for the VB trait using all markers and only a priori GWAS-associated markers across the three Research Stations. **A.** MSE in the training partition using all markers in Motilonia. **B.** MSE in the testing partition using all markers in Motilonia. **C.** MSE in the training partition using associated markers in Motilonia. **D.** MSE in the testing partition using associated markers in Motilonia. **E.** MSE in the training partition using all markers in Carmen de Bolivar **F.** MSE in the testing partition using all markers in Carmen de Bolivar. **G.** MSE in the training partition using associated markers in Carmen de Bolivar. **H.** MSE in the testing partition using associated markers in Carmen de Bolivar.

References

1. Burbano-Erazo, E.; León-Pacheco, R.I.; Cordero-Cordero, C.C.; López-Hernández, F.; Cortés, A.J.; Tofiño-Rivera, A.P. Multi-Environment Yield Components in Advanced Common Bean (*Phaseolus vulgaris* L.) × Tepary Bean (*P. acutifolius* A. Gray) Interspecific Lines for Heat and Drought Tolerance. *Agronomy* **2021**, *11*, 1978. https://doi.org/10.3390/agronomy11101978.
2. Montesinos-López, O.A.; Montesinos-López, A.; Pérez-Rodríguez, P.; Barrón-López, J.A.; Martini, J.W.R.; Fajardo-Flores, S.B.; Gaytan-Lugo, L.S.; Santana-Mancilla, P.C.; Crossa, J. A Review of Deep Learning Applications for Genomic Selection. *BMC Genomics* **2021**, *22*, 19. https://doi.org/10.1186/s12864-020-07319-x.
