# Supplementary figures and images for: Genomic Prediction of Adaptation in Common Bean (Phaseolus vulgaris L.) × Tepary Bean (P. acutifolius A. Gray) Hybrids
Source: Int J Mol Sci. 2025 Jul 30;26(15):7370. doi: 10.3390/ijms26157370 (PMC12347012; doi:10.3390/ijms26157370)

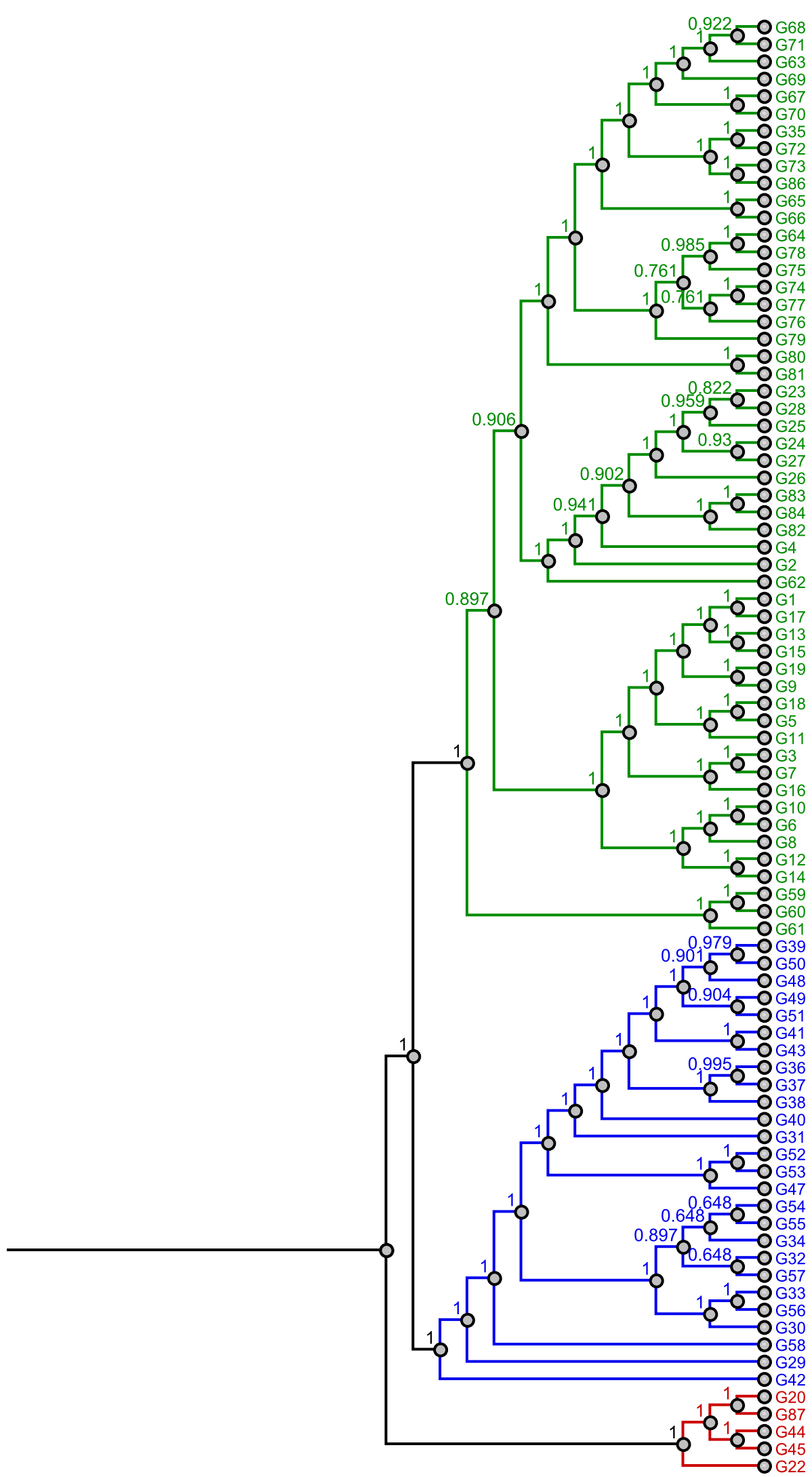

Supplement: Supplementary file 1 [file ijms-26-07370-s001.zip › FigureS1.pdf]

## Research Station Motilonia

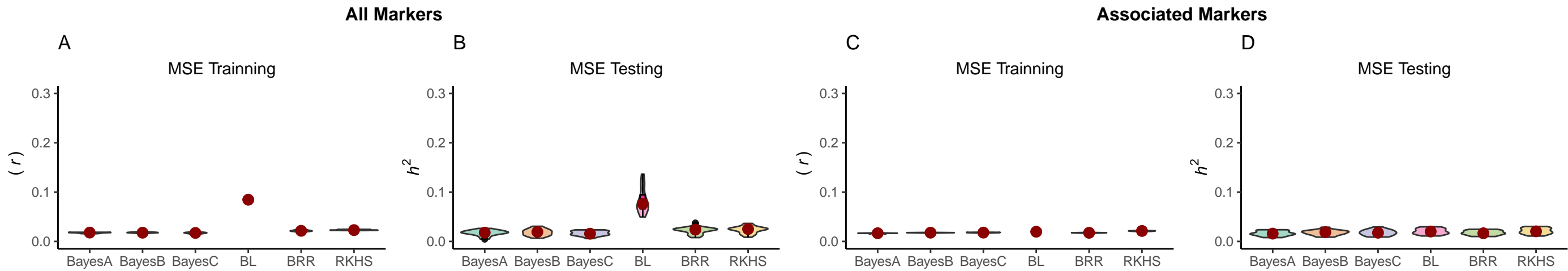

## Research Station Turipana

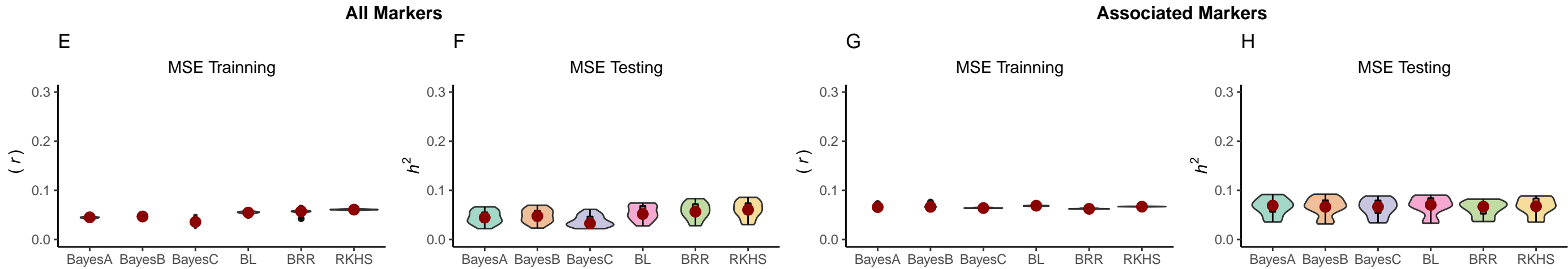

## Research Station Carmen de Bolivar

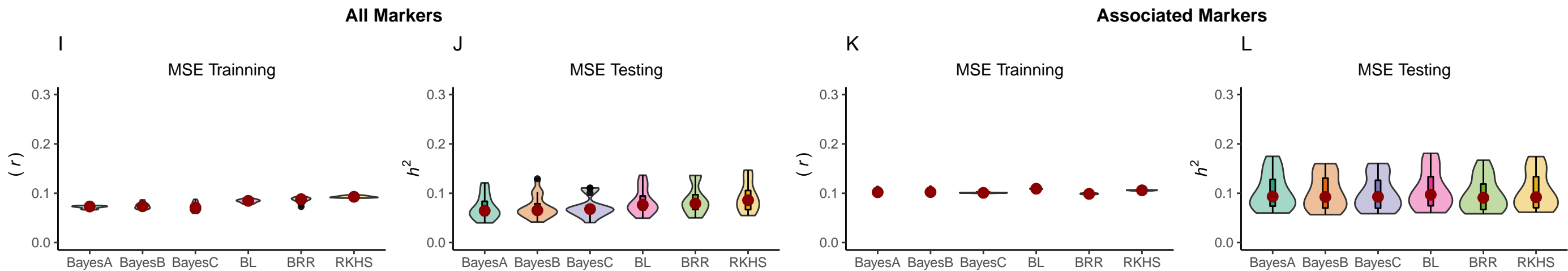

Supplement: Supplementary file 1 [file ijms-26-07370-s001.zip › FigureS10.pdf]

## Research Station Motilonia

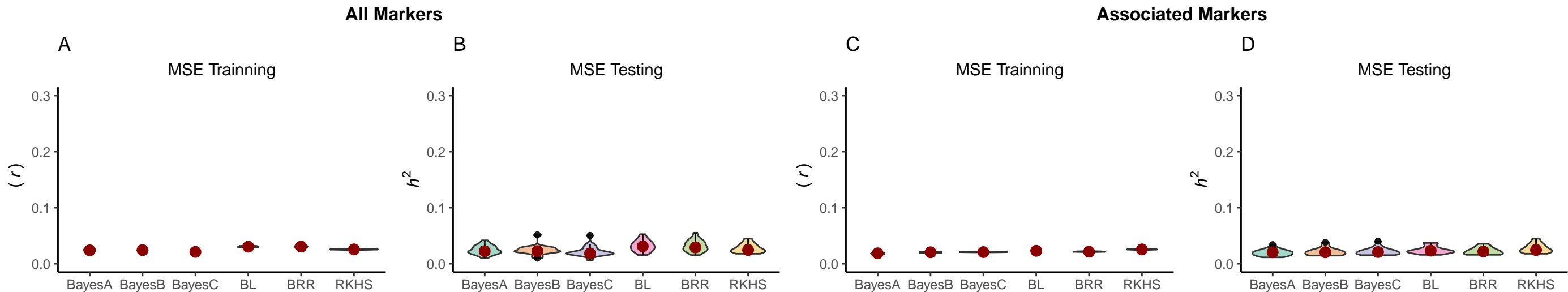

## Research Station Carmen de Bolivar

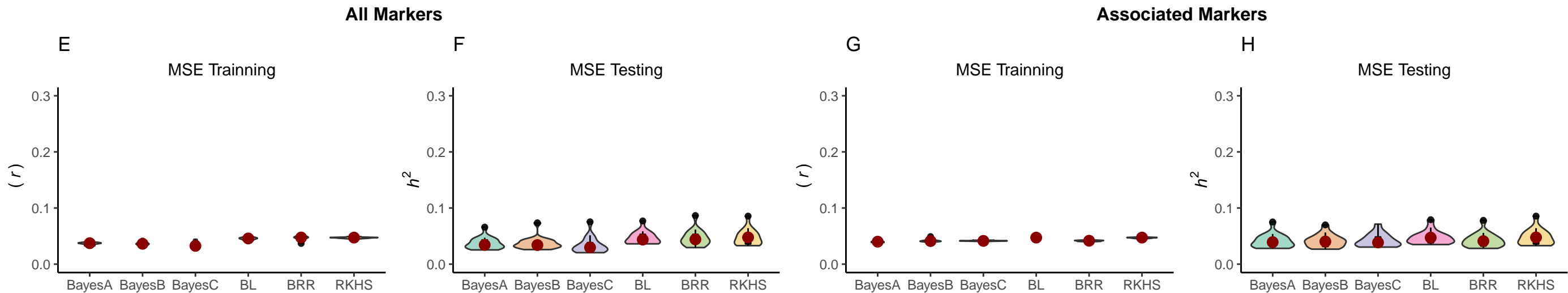

Supplement: Supplementary file 1 [file ijms-26-07370-s001.zip › FigureS11.pdf]

**A**

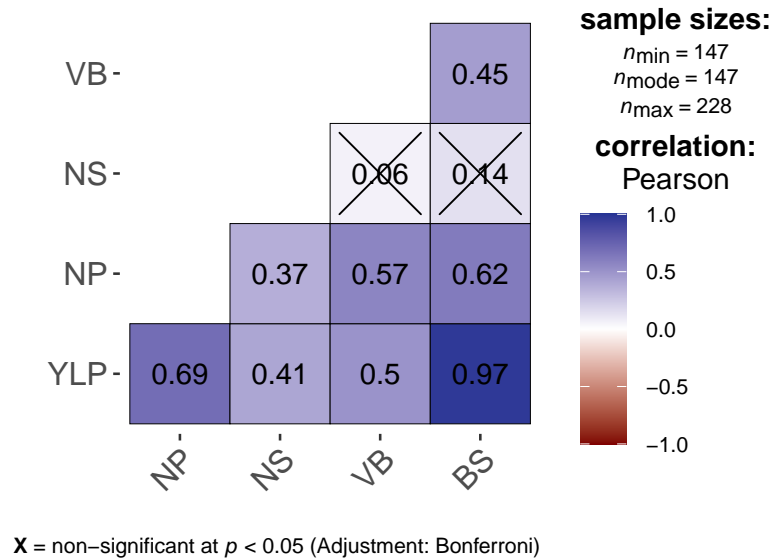

**B**

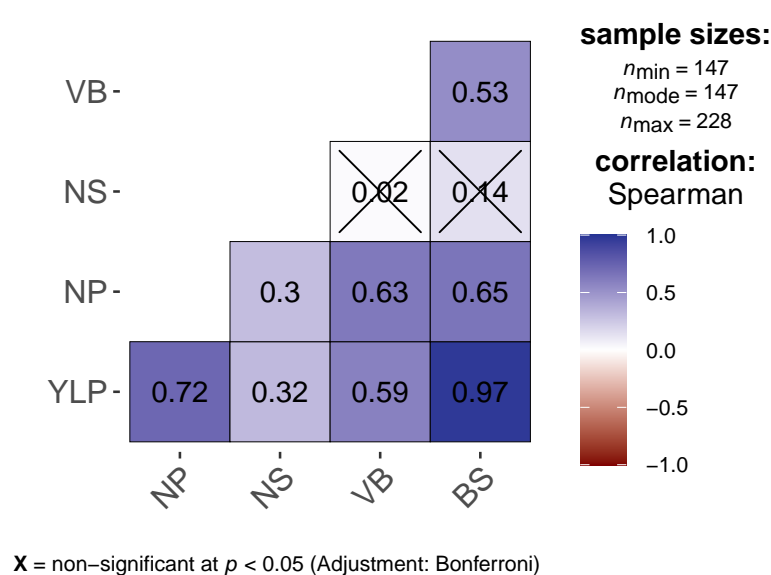

**C**

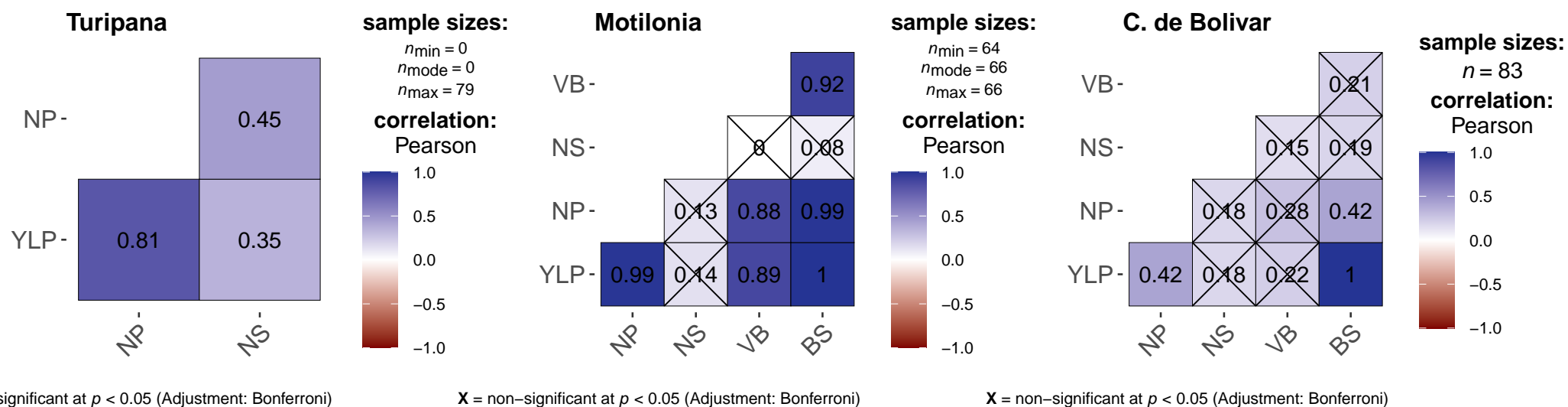

**D**

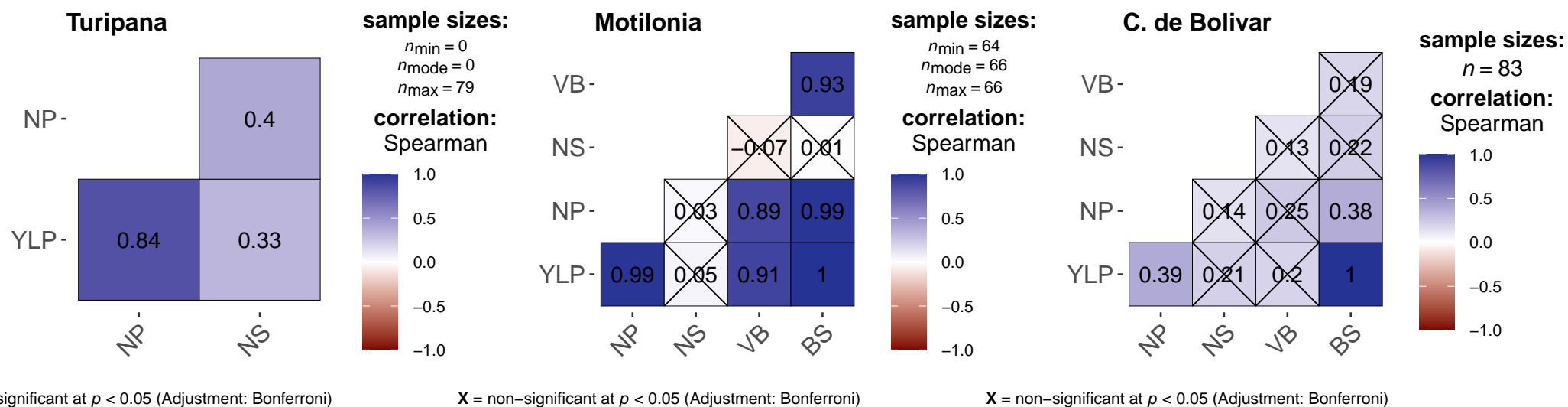

Supplement: Supplementary file 1 [file ijms-26-07370-s001.zip › FigureS2.pdf]

## Research Station Motilonia

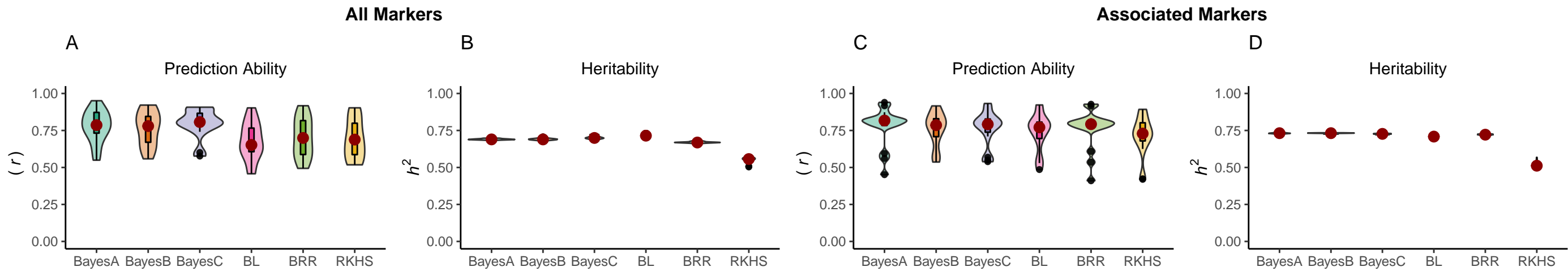

## Research Station Turipana

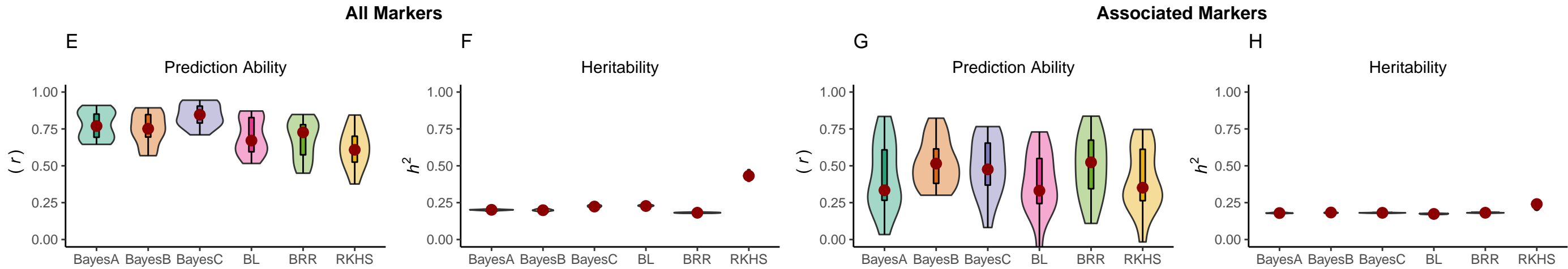

## Research Station Carmen de Bolivar

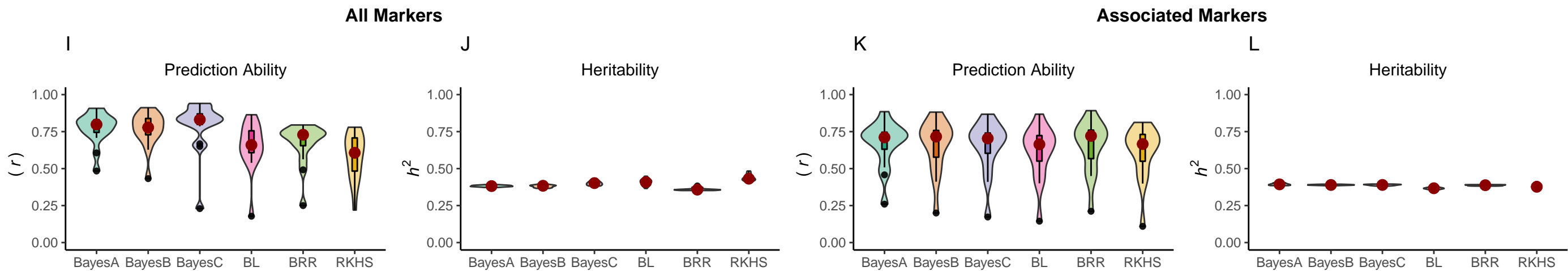

Supplement: Supplementary file 1 [file ijms-26-07370-s001.zip › FigureS4.pdf]

## Research Station Motilonia

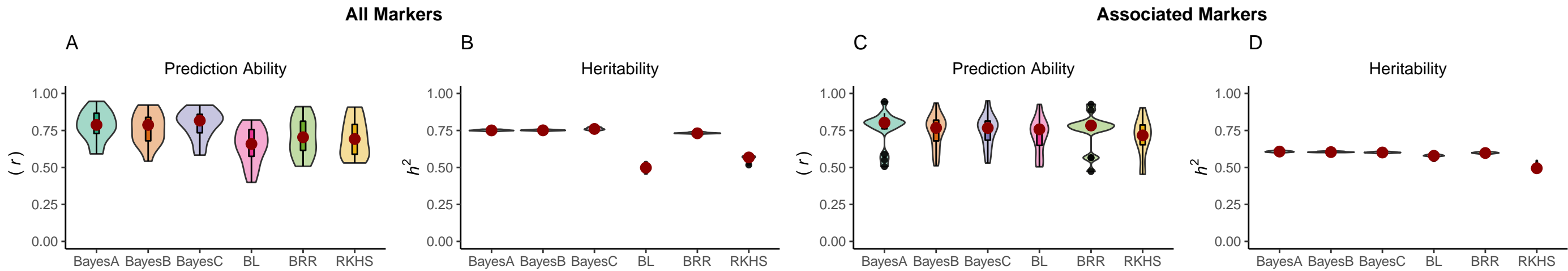

## Research Station Turipana

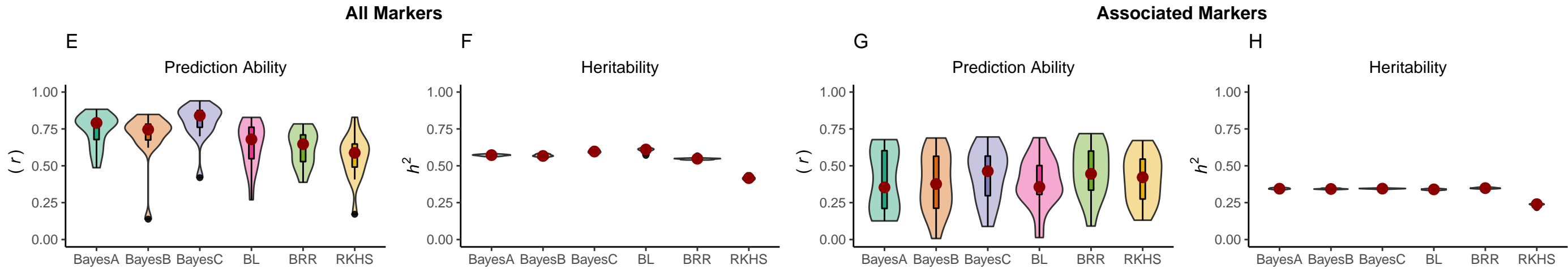

## Research Station Carmen de Bolivar

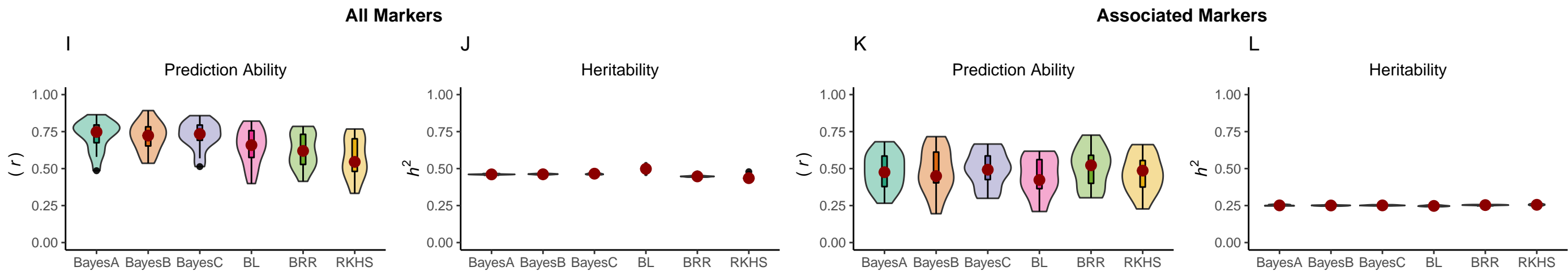

Supplement: Supplementary file 1 [file ijms-26-07370-s001.zip › FigureS5.pdf]

## Research Station Motilonia

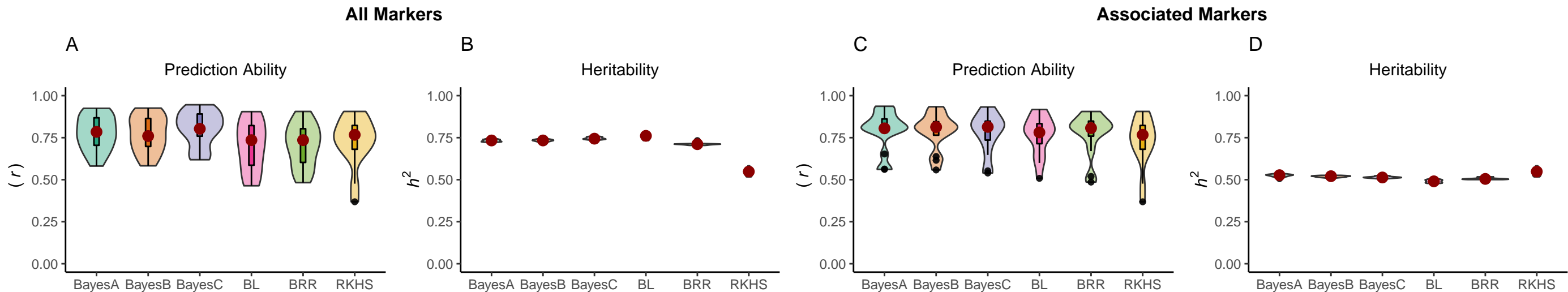

## Research Station Carmen de Bolivar

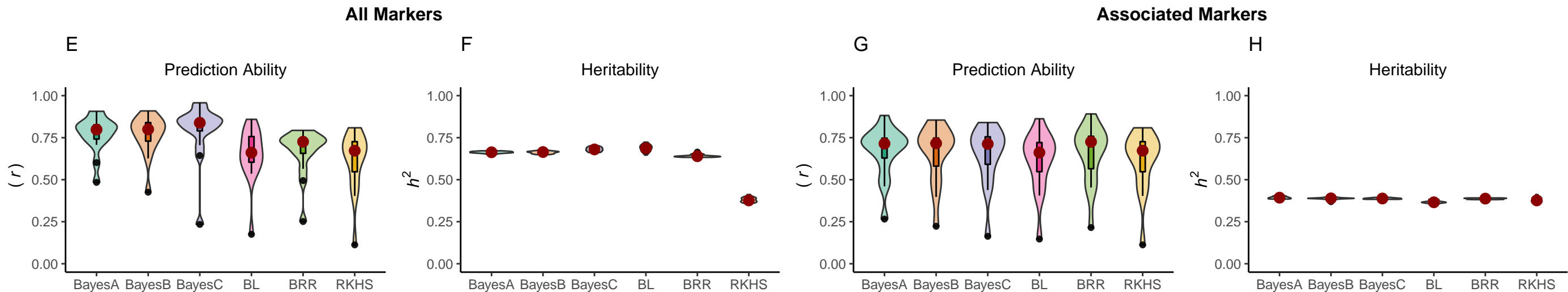

Supplement: Supplementary file 1 [file ijms-26-07370-s001.zip › FigureS6.pdf]

## Research Station Motilonia

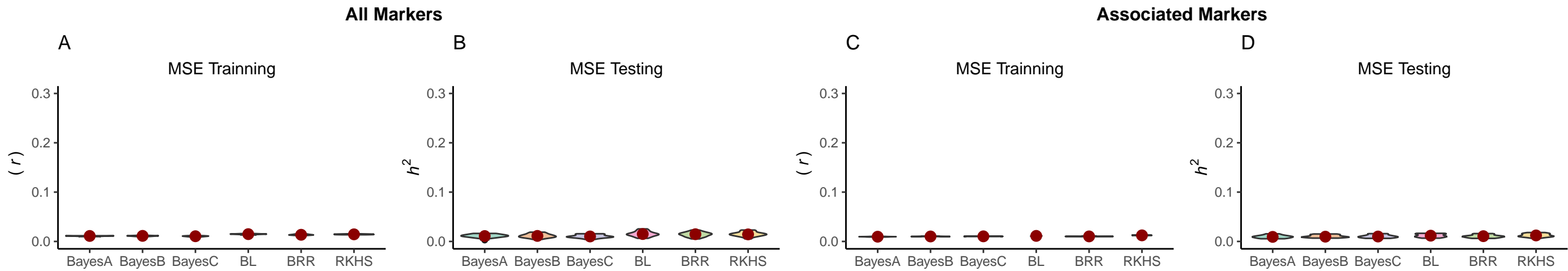

## Research Station Turipana

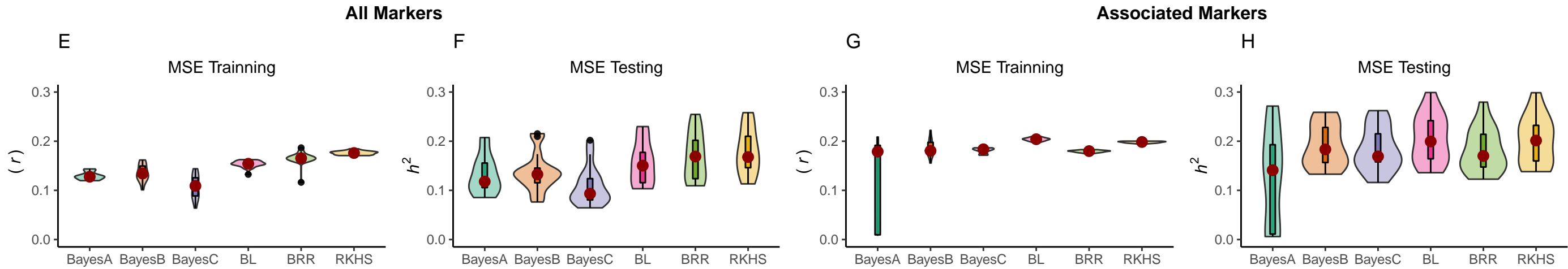

## Research Station Carmen de Bolivar

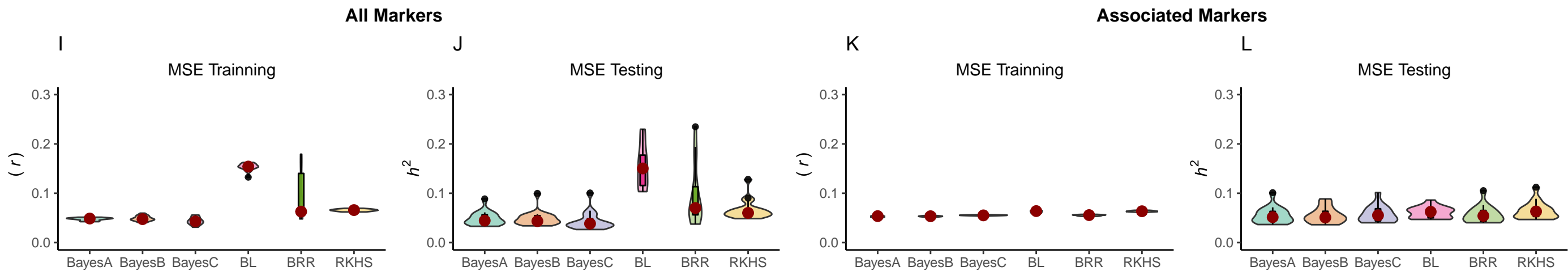

Supplement: Supplementary file 1 [file ijms-26-07370-s001.zip › FigureS8.pdf]

## Research Station Motilonia

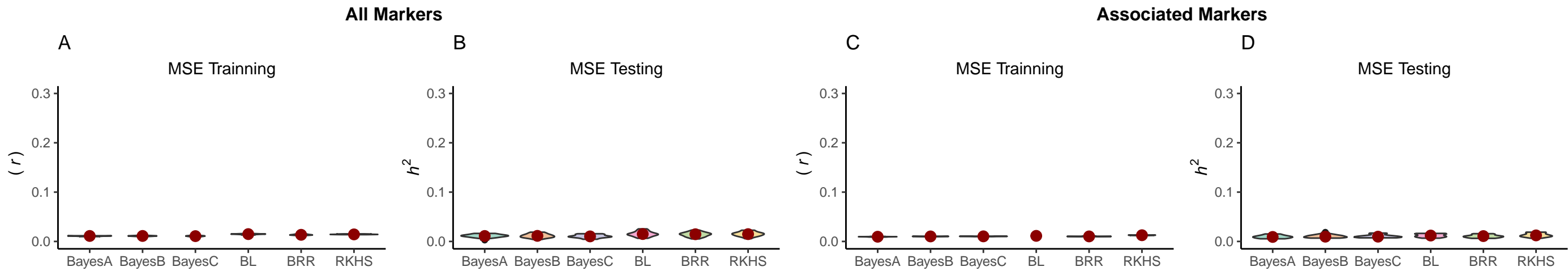

## Research Station Turipana

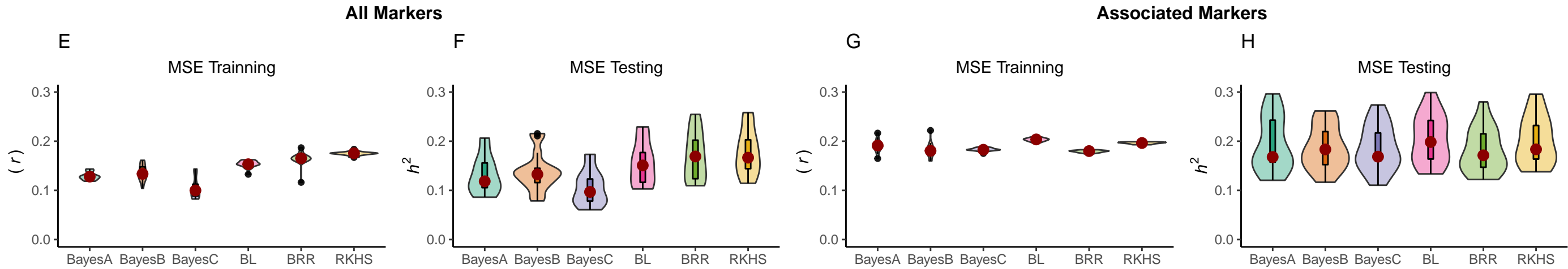

## Research Station Carmen de Bolivar

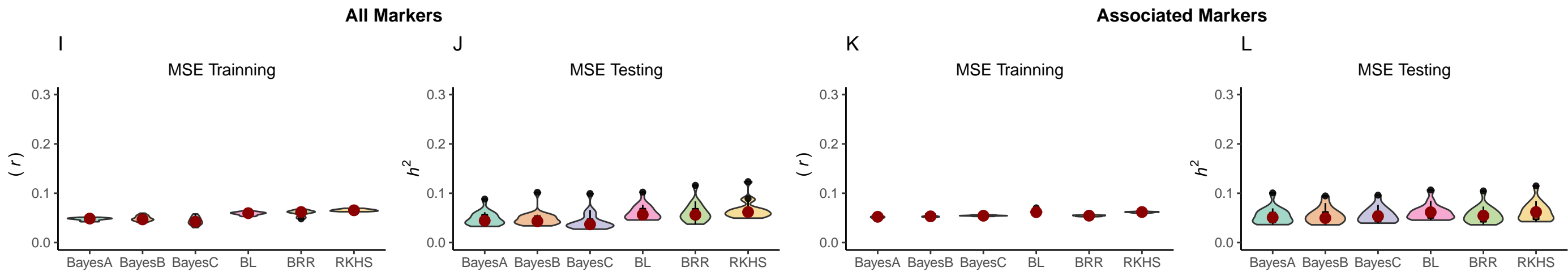

Supplement: Supplementary file 1 [file ijms-26-07370-s001.zip › FigureS9.pdf]
